# Allosteric regulation of substrate channeling: *Salmonella typhimurium* tryptophan synthase

**DOI:** 10.3389/fmolb.2022.923042

**Published:** 2022-09-12

**Authors:** Rittik K. Ghosh, Eduardo Hilario, Chia-en A. Chang, Leonard J. Mueller, Michael F. Dunn

**Affiliations:** ^1^ Department of Biochemistry, University of California, Riverside, Riverside, CA, United States; ^2^ Department of Chemistry, University of California, Riverside, Riverside, CA, United States

**Keywords:** tryptophan synthase, allostery, regulation, L-tryptophan, indole, catalysis, channeling

## Abstract

The regulation of the synthesis of L-tryptophan (L-Trp) in enteric bacteria begins at the level of gene expression where the cellular concentration of L-Trp tightly controls expression of the five enzymes of the Trp operon responsible for the synthesis of L-Trp. Two of these enzymes, trpA and trpB, form an αββα bienzyme complex, designated as tryptophan synthase (TS). TS carries out the last two enzymatic processes comprising the synthesis of L-Trp. The TS α-subunits catalyze the cleavage of 3-indole D-glyceraldehyde 3′-phosphate to indole and D-glyceraldehyde 3-phosphate; the pyridoxal phosphate-requiring β-subunits catalyze a nine-step reaction sequence to replace the L-Ser hydroxyl by indole giving L-Trp and a water molecule. Within αβ dimeric units of the αββα bienzyme complex, the common intermediate indole is channeled from the α site to the β site via an interconnecting 25 Å-long tunnel. The TS system provides an unusual example of allosteric control wherein the structures of the nine different covalent intermediates along the β-reaction catalytic path and substrate binding to the α-site provide the allosteric triggers for switching the αββα system between the open (T) and closed (R) allosteric states. This triggering provides a linkage that couples the allosteric conformational coordinate to the covalent chemical reaction coordinates at the α- and β-sites. This coupling drives the α- and β-sites between T and R conformations to achieve regulation of substrate binding and/or product release, modulation of the α- and β-site catalytic activities, prevention of indole escape from the confines of the active sites and the interconnecting tunnel, and synchronization of the α- and β-site catalytic activities. Here we review recent advances in the understanding of the relationships between structure, function, and allosteric regulation of the complex found in *Salmonella typhimurium.*

## Introduction and background

### Regulation of L-Trp synthesis

**FIGURE 1:**
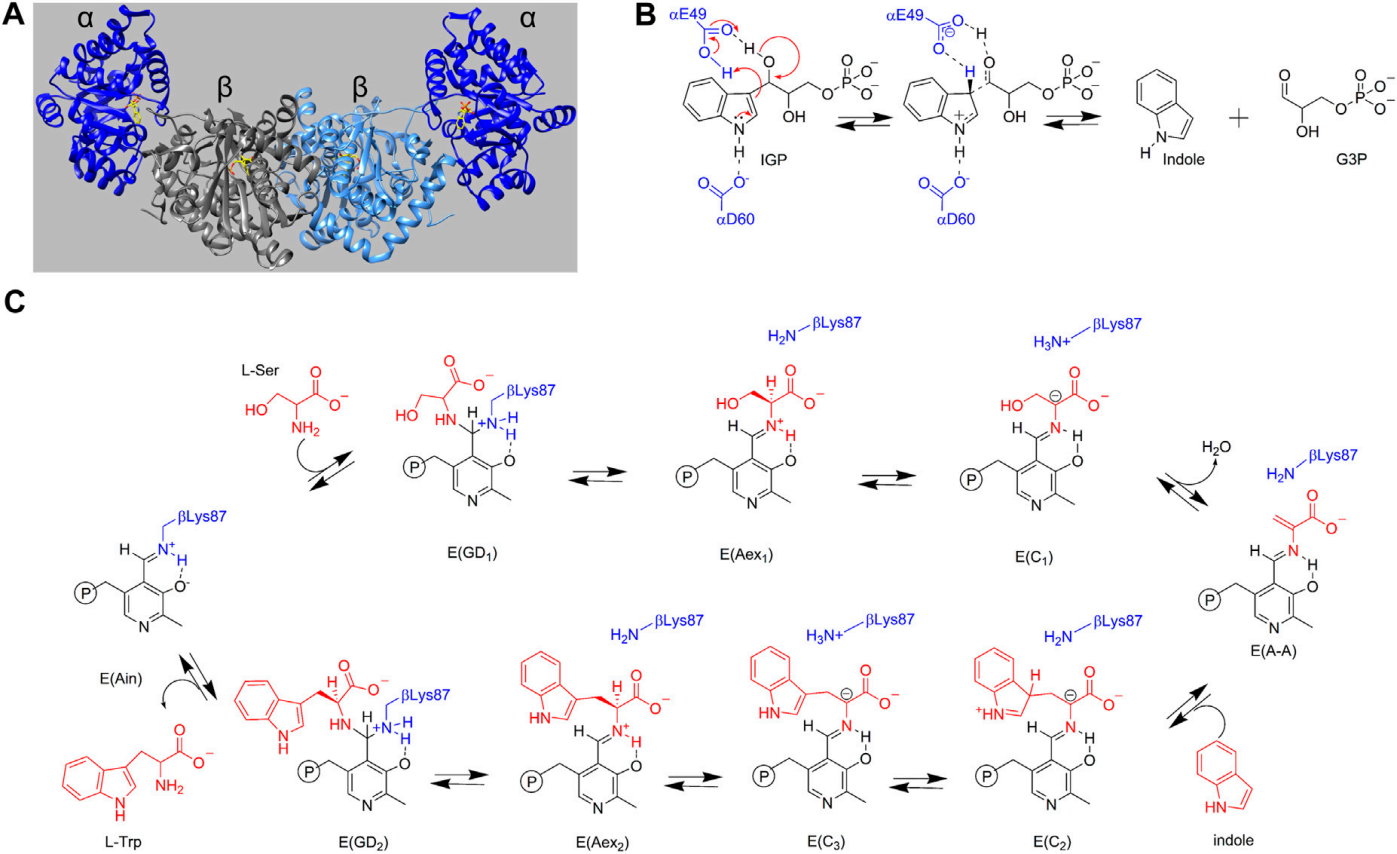
**(A)** Three-dimensional structural representation of the α_2_β_2_ tryptophan synthase hetero-tetrameric multienzyme complex from *S. typhimurium*. α-Subunits dark blue, β-subunits gray and light blue. The substrate for the α site, 3-indole D-glyceraldehyde 3′-phosphate, and the PLP cofactor as the internal aldimine covalently attached to βLys87 at the β-site are shown with yellow carbons. PDB ID: 2RHG. **(B)** α-Reaction with catalytic residues αGlu49 and αAsp60 shown in blue. **(C)** β-Reaction with the reacting substrates L-Ser and indole and product L-Trp shown in red. Catalytic residue βLys87 is shown in blue. PLP species are shown in black and reacting substrate species are shown in red.

**FIGURE 2:**
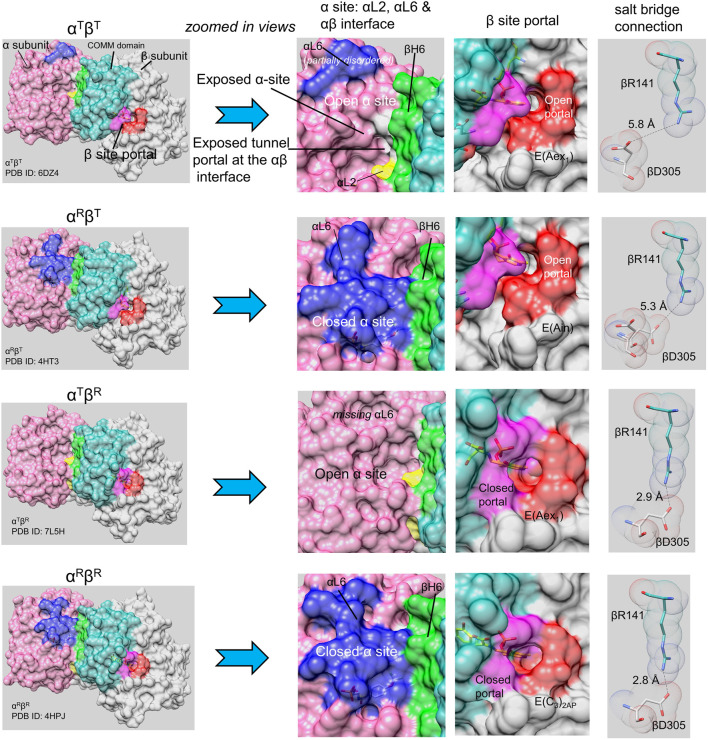
The TS allostery model consists of two subunit conformations, T and R, and four quaternary states, α^T^β^T^, α^R^β^T^, α^T^β^R^ and α^R^β^R^ (Niks et al., 2013). Surface models are shown for the four quaternary states (left column) for each heterodimeric unit. The left column provides an overview of each quaternary state: each panel shows the α-subunit in light purple with loop αL2 (residues 53–60) yellow, loop αL6 (residues 179–193) blue; the β-subunit is shown in gray with the COMM domain (residues 102–189) in light blue-green and helix βH6 in green. The β-site portal is shown in dark purple (COMM domain residues) and orange. The central two columns show expanded views of the α- and β-subunits focusing on the catalytic sites, the α-β subunit interface, and with the portals into the interconnecting tunnel. Ligands bound to the α- and β-sites are shown as sticks. The last column shows structural detail for βArg141 and βAsp305 in each quaternary state; when the β-subunit is closed, these residues form an H-bonded salt bridge. Notice that the loop αL6 residues are either partially missing or completely missing in the α^T^ conformation, and that the β-subunit portal switches between open and closed states depending on whether the β-subunit is in the β^T^ or β^R^ conformation. Images rendered in PyMol (Schrodinger, 2010).

**FIGURE 3:**
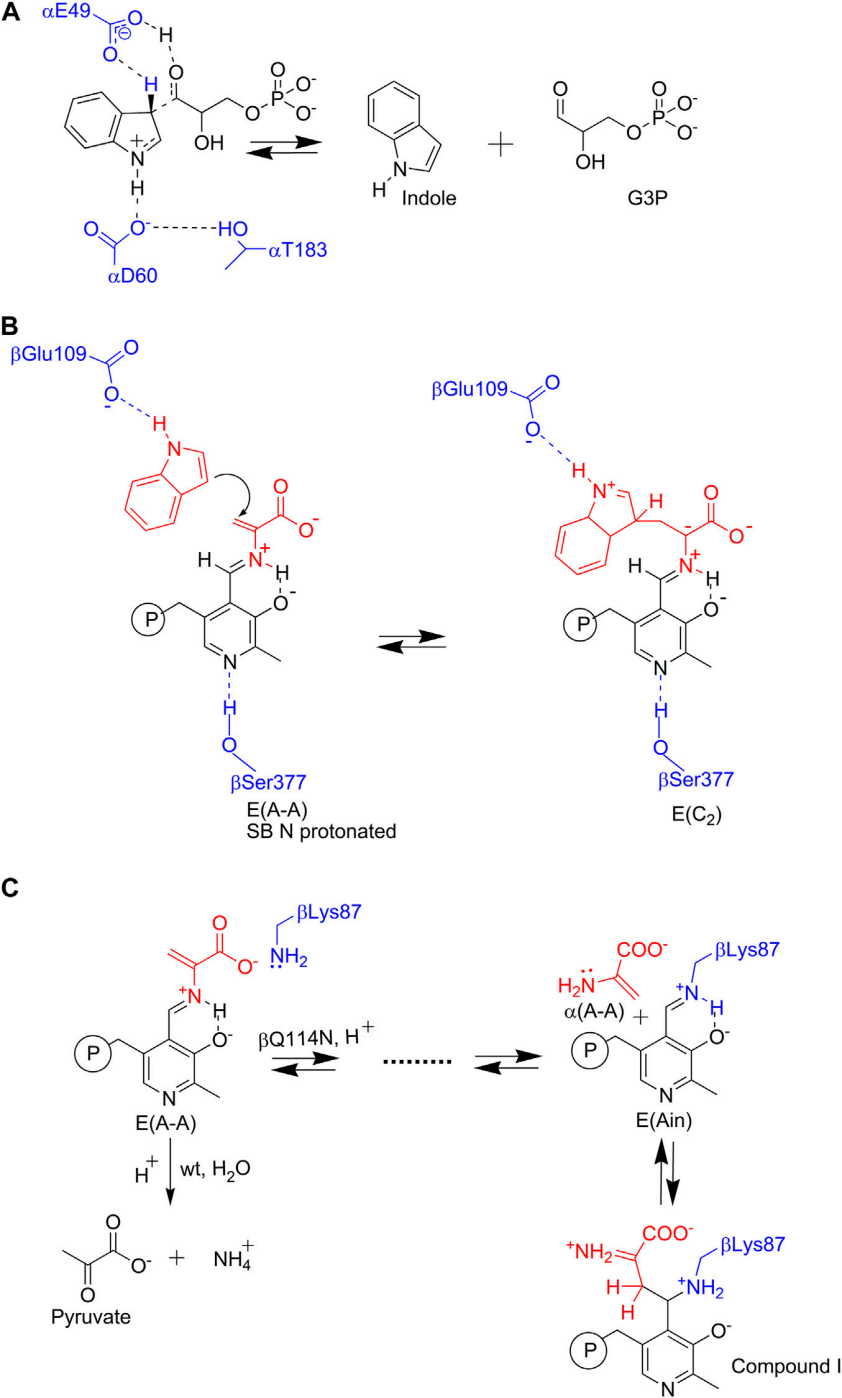
Nucleophilic reactions catalyzed by TS. In the α- and β-reactions (Figures 1B,C), the C-C bond scission and bond formation steps require the reacting indole ring to participate as an enamine wherein the relatively electron rich C3 makes a nucleophilic attack **(A)** on a proton (in the α-reaction), or **(B)** on the α-aminoacrylate intermediate Cβ (in the β-reaction). Because the indole ring system is a relatively weak nucleophile, TS has evolved a mechanism for enhancing the nucleophilicity of the indole ring C3 through stabilization of the zwitterionic transition states for protonation **(A)** or C-C bond formation **(B)** *via* Coulombic interaction of the carboxylate ion of an active site residue. At the α-site, it is postulated that the hydroxyl of αL6 loop residue αThr183 stabilizes this interaction through a hydrogen bond to the αAsp60 carboxylate. This interaction only occurs in the αR state when loop αL6 is well ordered (Kulik et al., 2005). In the β-reaction when indole binds to its sub-site, the βGlu109 carboxylate hydrogen bonds to the N1 proton of the indole ring and thus stabilizes the transition state for C-C bond formation in the nucleophilic attack of C3 on the α-aminoacrylate Cβ. **(C)** The εNH_2_ of βLys87 initiates a deleterious side reaction by making a nucleophilic attack at the PLP C4′ resulting in the release of the three-carbon enamine, α-aminoacrylate in the βQ114N mutant (Kulik et al., 2005). This powerful nucleophile then attacks C4′ of E(Ain) forming a C-C bond and yields a tightly bound PLP derivative that inactivates the β-site. This complex has a β^R^ conformation.

The ability to synthesize L-Trp (**1**) is essential for many organisms including enteric bacteria, yeasts, molds, and plants. L-Trp synthesis is tightly regulated in enteric bacteria, and this control begins at the level of gene expression (Yanofsky, 1955; Yanofsky, 1981; Yanofsky, 1987). In these bacteria, the trp operon is regulated by the *in vivo* concentration of L-Trp. Binding of L-Trp to the trp repressor protein stabilizes the formation of the trp operon-trp repressor complex, effectively blocking expression of the operon and hence shutting down the synthesis of L-Trp. When the cellular concentration of L-Trp becomes too low to bind to the trp repressor, the trp repressor dissociates from the trp operon, triggering the expression of the five proteins responsible for L-Trp synthesis, trpE, trpD, trpC, trpB, and trpA. The enzymes expressed by trpA and trpB form an αββα bienzyme complex (Figure 1A) that carries out the last two steps in the synthesis of L-Trp (the α- and β-reactions) (Figures 1B,C). This complex is designated here as tryptophan synthase, TS. The TS α-subunit has the canonical (βα)_8_ TIM barrel fold (Hyde et al., 1988), first observed in triosephosphate isomerase, while the β-subunit has an unusual fold structure comprised of the COMM domain (β102—β189; Figures 1A, 2) (Schneider et al., 1998) and a scaffolding (β1–β101 and β190—β395) that supports the COMM domain and contributes residues to the 25–30 Å long tunnel that connects the α- and β-sites (Figures 3A,B). While Kirschner et al. (1975) were the first to report evidence for ligand induced interactions between the α- and β-sites, Drewe and Dunn (1986) were the first to explicitly recognize that tryptophan synthase catalysis of L-Trp synthesis is subject to control via ligand mediated allosteric interactions transmitted between the α- and β-subunits.

At this juncture, structural and mechanistic investigations of TS-allostery show that the TS α_2_β_2_ bienzyme complex is a multifaceted, highly nuanced molecular machine. This machine utilizes substrate channeling, pyridoxal phosphate, a monovalent cation effector, a hydrophobic nanotube and heterotropic allosteric site-site interactions, to regulate and synchronize the activities of the αβ-subunit pairs to achieve the efficient synthesis of L-tryptophan.

### Catalysis in the α_2_β_2_ TS complex

In Escherichia *coli* and *Salmonella enterica* serovar *Typhimurium*, assembly of the α and β subunits into the α_2_β_2_ TS complex is essential for full activity (Yanofsky and Crawford, 1972; Creighton, 1970; Miles, 1979; Bahar and Jernigan, 1999; Miles, 2001; Raboni et al., 2009; Kulik et al., 2005; Dunn, 2012; Barends et al., 2008a; Miles, 2013; Maria-Solano et al., 2019; Sakhrani et al., 2020; Buller et al., 2015). The isolated α-subunit shows a catalytic activity diminished by ∼ 1,000- to 3,000-fold while compared to the holo α_2_β_2_ complex the activity of the PLP-requiring β_2_ dimer, is diminished by ∼100 fold (Yanofsky and Crawford, 1972; Miles, 1979; Miles, 2001; Kulik et al., 2005; Barends et al., 2008a; Dunn, 2012; Miles, 2013; Buller et al., 2015). The α-subunits catalyze the cleavage of substrate 3-indole D-glyceraldehyde 3′-phosphate (IGP), yielding indole and D-glyceraldehyde 3-phosphate (G3P, **6**) (Figure 1B, the α-reaction) Yanofsky and Crawford, 1972; Miles, 1979; Miles, 2001; Barends et al., 2008a; Kulik et al., 2002), while the β-subunits catalyze replacement of the L-Ser (**7**) β-hydroxyl by indole yielding L-Trp and a water molecule (Figure 1C, the β-reaction) (Yanofsky and Crawford, 1972; Miles, 1979; Drewe, and Dunn, 1985, 1986; Dunn, 2012; Miles, 2013). During the catalytic cycle, indole formed at the α-site is transferred to the β-site via the 25 Å-long, tunnel (Creighton, 1970; Yanofsky and Crawford, 1972; Matchett, 1974; Miles, 1979; Hyde et al., 1988; Dunn et al., 1990; Brzovic P. S. et al., 1992; Miles, 2001; Raboni et al., 2009; Miles, 2013; Hilario et al., 2016; Teixeira et al., 2020). This channeling of the common intermediate, indole, is a key feature of the allosteric control mechanism for the synthesis of L-Trp (Dunn et al., 1990; Miles, 2001; Raboni, et al., 2009; Dunn, 2012; Miles, 2013; Hilario et al., 2016; Teixeira et al., 2020). Within α_2_β_2_, αβ dimers form allosteric units that work independently of each other while the allosteric interactions within each αβ unit are essential to the efficient synthesis of L-Trp (Dunn, 2012; Niks et al., 2013; Ghosh et al., 2021).

### Recent structure-function studies

The first x-ray structures of TS were reported by Hyde et al. (1988) (viz. Figures 1A, 2). During the past 20 years a relatively large number of TS structures have been deposited in the protein data bank (PDB) (Berman et al., 2000). Most of these are structures of the *Salmonella typhimurium* bienzyme in complex with substrates, and with a variety of substrate analogues and/or covalent intermediates bound to the α- and β-sites (viz. Figure 2). These structures provide a rich source of information relevant to 1) the chemical structures of intermediates, 2) the reaction mechanisms within the catalytic cycles of the α- and β-reactions (Figures 1B,C), and 3) the structures of the conformational states (Figure 2) and allosteric transitions of the α- and β-subunits during these reaction cycles. The TS literature is replete with physical biochemical investigations of the α- and β-reactions (Yanofsky and Crawford, 1972; Miles, 1979; Miles, 2001; Raboni et al., 2009; Dunn, 2012; Miles, 2013). Recent publications include a variety of solution UV/Vis rapid kinetic studies (Harris et al., 2005; Ngo et al., 2007a, b; Dierkers et al., 2009; Ghosh et al., 2021; Phillips and Harris, 2021) and NMR studies employing ligands with ^19^F (Niks et al., 2013) and ^17^O probes (Young et al., 2016). Solid state magic angle spinning ssNMR investigations of TS complexes of substrate and substrate analogues and with ^13^C and ^15^N enriched PLP (McDowell et al., 1995; Lai et al., 2011; Caulkins et al., 2014, 2016; Holmes et al., 2022), and β-subunits enriched with ^15^N-Lys residues (Caulkins et al., 2014) and molecular dynamics studies (Huang et al., 2016; Maria-Solano et al., 2019; O’Rourke et al., 2019) have become especially important for establishing the predominant protonation states and tautomeric states of PLP intermediates in the β-reaction.

### Catalysis at the α- and β-Sites

**FIGURE 4:**
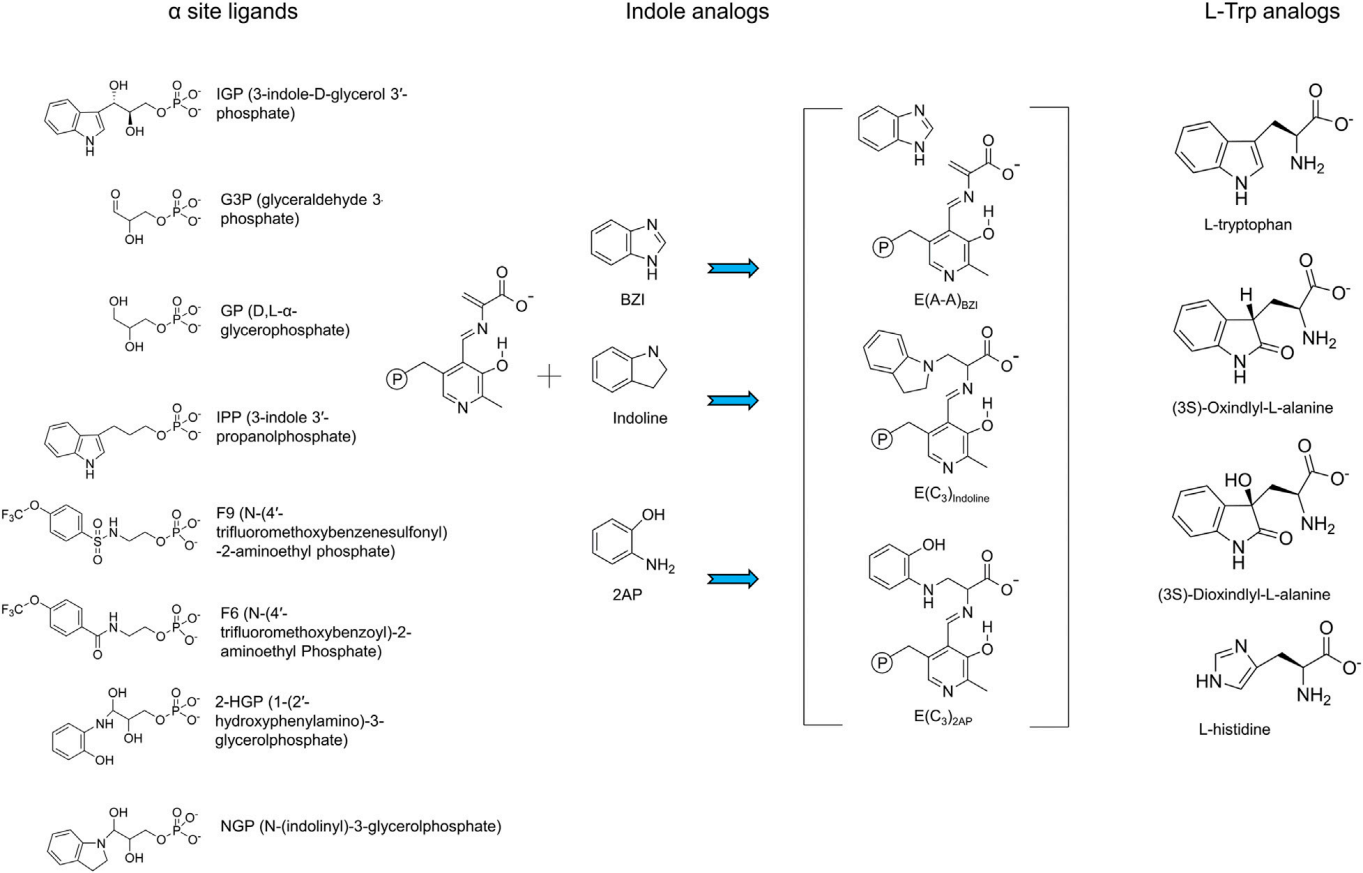
Summary of substrates and substrate analogues.

Catalysis at the α-site involves formation of a complex wherein the reacting substrate, IGP, undergoes a reverse aldolytic cleavage reaction catalyzed by two acid-base catalytic groups, αGlu49 and αAsp60 (Figure 3A). The pioneering site-directed mutagenesis work by Edith Miles provided the first evidence that the conversion of indole into an effective nucleophile requires the coupling of a charge-stabilizing interaction between a side chain carboxylate and the indole N-1, both in the α-reaction (αAsp60) and in the β-reaction (βGlu109) (Figures 3A,B) (Yutani et al., 1987; Miles et al., 1988; Nagata et al., 1989; Brzovic P. S. et al., 1992). Catalysis at the β-site involves the interconversion of at least nine covalent intermediates (Figure 1B) (Yanofsky and Crawford, 1972; Miles, 1979; Miles, 2001; Raboni et al., 2009; Dunn, 2012; Drewe and Dunn, 1985, 1986; Ngo et al., 2007b; Lai et al., 2011; Phillips and Harris, 2021). Recent mechanistic studies conclusively show that the εNH_2_ group of βLys87 plays essential roles in the formation of the internal and external aldimines of substrates L-Ser and L-Trp (Figure 1B ) and provides the acid-base catalysis for all the various proton transfers involved in the interconversion of gem diamines with internal aldimines and external aldimines and with carbanionic intermediates along the catalytic path (Caulkins et al., 2014, 2016; Huang et al., 2016; Holmes et al., 2022). The carboxylate of βGlu109 participates in a columbic charge-charge, H-bonding interaction that stabilizes development of a partial positive charge at the indole ring N-1 nitrogen as the C-C bond is formed between the indole C_3_ and the C_β_ of the α-aminoacrylate intermediate (Figure 3B) (Brzovic P. S. et al., 1992; Holmes et al., 2022). This interaction is essential to the chemical activation of weakly nucleophilic indole for the enamine attack via C_3_ (Figure 3B). Notice that, in contrast to the classical view in which carbanionic intermediates are formed and stabilized as quinonoidal species in the catalytic cycles of many PLP-requiring enzymes, the TS PLP ring N remains unprotonated throughout the entire β-reaction cycle and therefore cannot form canonical quinonoid structures (Caulkins et al., 2016; Holmes et al., 2022) (Figure 1C). Nevertheless, carbanionic species with the negative charge delocalized over the C_α_, Schiff base N, C_4’_, C_4_, C_3_ and O_3_ atoms of the PLP ring scaffolding give intermediates that are quasi-stable (Dunn, 2012; Caulkins et al., 2016) and provide spectroscopic analogues of the transiently formed L-Ser and L-Trp carbanions detected in the β-reaction (Drewe and Dunn, 1985; 1986; Roy et al., 1988a, b; Barends et al., 2008a, b; Caulkins et al., 2016; Holmes et al., 2022; Ghosh et al., 2021; Phillips and Harris, 2021) (organic structures are summarized in Figure 4).

Blumenstein et al. (2007), observed that the mutation of βGln114 to Asn facilitates a side reaction wherein the εNH_2_ of βLys87 makes a nucleophilic attack at the PLP C4′ of E(A-A), giving E(Ain) and releasing α-aminoacrylate. This highly nucleophilic three-carbon enamine then reacts to form a new C-C bond with the E(Ain) PLP C4′ carbon (Figure 3C). This reaction progresses to give an inactivated TS derivative with a covalently modified PLP derivative bound to the β^R^ state (PDB ID: 2J9Y). The α-aminoacrylate side reaction has been reported for several other PLP-dependent enzymes that involve α-aminoacrylate Schiff base intermediates (Roise et al., 1984). It appears that bacterial systems have evolved an enzyme family, RidA, with the primary biological function of deaminating 3- and 4-carbon enamines to prevent similar side reactions from occurring in PLP-dependent enzymes (Flynn and Downs, 2013).

Atomistic MD simulations provide detailed insights into allosteric regulation, allosteric networks, and indole channeling (Fatmi et al., 2009). These MD studies sampled both open and partially closed conformations of the highly flexible α-subunit loop 6 (αL6) in the ligand-free and ligand bound states; the loop shifts to fully closed conformations when α site ligands are present. Post-MD analysis to compute energy and configuration entropy suggests that the fully closed conformations are induced by favorable protein-ligand interactions but are partly offset by configurational entropy loss (Fatmi et al., 2009). The αβ-dimeric unit stabilizes the substrate-protein conformation, which also forms new hydrogen bonds and lowers the conformation transition barrier to facilitate the conformation transition from an open/inactive form to a closed/active form (Fatmi and Chang 2010; O’Rourke et al., 2019). The allosteric motions regulate substrate catalysis, and the MD simulations identified interaction changes across the catalytic cycle of the α-reaction (Spyrakis et al., 2006; D’Amico et al., 2021; Bosken et al., 2022).

Although hydrogen atoms and water molecules may not directly contribute to allosteric regulation, MD and quantum mechanics studies suggest that the protonation states and conserved water molecules affect TS motions which are important for the catalytic process (Huang et al., 2016; Teixeira et al., 2019). Combined MD simulations and ancestral sequence reconstruction identify residues contributing to allosteric signal propagation in TS (Schupfner et al., 2020). In addition to classical MD simulations, enhanced sampling methods, such as steered MD simulation, have been applied to examine indole channeling between the α- and β-subunits to scrutinize interactions between indole and residues lining the channel (Zhang and Lazim, 2019).

### Protein allosteric states are comprised of ensembles

Recent literature on the nature of allosteric transitions and protein conformation states hypothesize that the T and R designations for the conformational states of allosteric protein systems as proposed by Monod, Wyman and Changeux and Koshland, Nemethy and Filmer (Monod et al., 1965; Kirschner et al., 1966; Koshland et al., 1966) are insufficiently nuanced and that the discussion of allosteric mechanism should be reframed within the context of protein conformational ensembles that are optimized via evolution to achieve control of biological function (Goodey and Benkovic, 2008; Feher et al., 2014; Motlagh et al., 2014; Nussinov and Tsai, 2014; Wei et al., 2016; Buchenberg et al., 2017; Guarnera and Berezovsky, 2019; Wodak et al., 2019; Verkhivker et al., 2020). Because the TS free energy landscapes are not yet well described by experiments, herein we apply the T and R nomenclature to the TS system while implicitly recognizing that the various noncovalent and covalent complexes detected in the TS system no doubt are comprised of ensembles which exist in equilibria around their native states and that the free energy barriers separating these ensembles comprise the free energy pathways described herein as the α- and β-reactions (Figures 1B,C) (Maria-Solano et al., 2019; Teixeira et al., 2020).

### Evidence for open and closed allosteric states in TS

As will be discussed in detail in following sections, the switching between open and closed subunit conformations plays a central role in the TS allosteric regulatory mechanism and is critically important to the efficient synthesis of L-Trp (Dunn et al., 1990; Kulik et al., 2005; Ngo et al., 2007b; Barends et al., 2008a; Lai et al., 2011; Niks et al., 2013; Caulkins et al., 2016; Ghosh et al., 2021). Many x-ray crystal structures of the open and closed states of TS are available in the Protein Data Bank archive (RCSB PDB) (Berman et al., 2000). With the high affinity IGP analogue F9 (**8**) bound to the α-site, the TS internal aldimine and the L-Ser external aldimine give complexes with closed α-subunits and open β-subunits (Niks et al., 2013; Ghosh et al., 2021) (viz, Figure 2). The F9 complexes with the α-aminoacrylate and carbanion intermediates almost always give structures where both subunits assume closed conformations (Ghosh et al., 2021). ^19^F NMR studies of the F9 complexes indicate these subunit conformations are also the predominate forms in solution (Niks et al., 2013). Our current working model for the TS allosteric mechanism is shown in Figure 2.

Mutations in the β-subunit can reverse the relative stabilities of β-subunit allosteric states. For example, the x-ray structure of the βGln114Ala mutant with α-aminoacrylate bound to the β-site and without ligand bound to the α-site shows an α^T^β^T^ complex (PDB ID: 7KQ9) and the structures of the E(Aex_1_) and E(Aex_2_) species formed with the βLys87Thr mutant give α^R^β^R^ complexes (Rhee et al., 1997).

Open and closed conformations of the TS subunits here are designated as T-state and R-state in conformity with early allosteric nomenclature (Monod et al., 1965; Niks et al., 2013; Ghosh et al., 2021). The designation of TS subunit conformations as either open or closed here has its origins in solution rapid kinetic experiments which demonstrate that the α- and β-subunits switch between conformations wherein ligands rapidly bind and dissociate, i.e., open conformations (T-state), and conformations wherein ligands slowly bind and dissociate, i.e., closed conformations (R-state) (Dunn et al., 1990; Harris et al., 2002; 2005; Ngo et al., 2007a,b; Ghosh et al., 2021). The x-ray structure database (Berman et al., 2000) confirms that TS switches between open and closed states in response to ligand binding at the α-site and to the covalent state of intermediates bound to the β-site (Figure 2) (Kulik et al., 2005; Ngo et al., 2007a, b; Barends et al., 2008a; Lai et al., 2011; Niks et al., 2013; Caulkins et al., 2016; Ngo et al., 2007a, b; Ghosh et al., 2021). Within α_2_β_2_, the switch of the α-subunit to the closed conformation when E(A-A) is formed activates the α-site by ∼30-fold while the β-subunit is activated about 10-fold (Anderson et al., 1991; Brzovic P. S. et al., 1992; Leja et al., 1995; Webber-Ban et al., 2001; Ngo et al., 2007b).

### Substrate channeling

The channeling of small molecules *via* tunnels within macromolecular assemblages is a key strategy in biological systems for the selective transfer of small molecules and ions among cellular compartments and across cell membranes (Dunn et al., 1990; Dunn et al., 2008; Shaffer et al., 2009; Galdiero et al., 2012; Ziervogel and Roux, 2013; Baker and Baldus, 2014; Horn et al., 2014; Aryal et al., 2015; Hilario, et al., 2016). Typically, the macromolecular protein structures responsible for these transfers form channels which achieve high selectivity for the transferred molecule or ion by acting as molecular filters that restrict passage through the channel based on charge, molecular cross-section, and hydrophobicity (Davis, 1967; Holden et al., 1998; Miles et al., 1999; Amaro et al., 2003; Amaro and Luthey-Schulten, 2004; Raushel et al., 2003; Amaro et al., 2005; Friedrich, 2014). Channeling also is an important phenomenon for certain enzyme assemblages within some metabolic pathways (Raushel et al., 2003). Substrate channeling among enzyme complexes can play important roles within the cell in preventing deleterious side reactions of labile small molecules, enhance catalytic efficiency, and prevent the loss of hydrophobic small molecules into hydrophobic environments (Dunn et al., 2008; Hilario et al., 2016).

**FIGURE 5:**
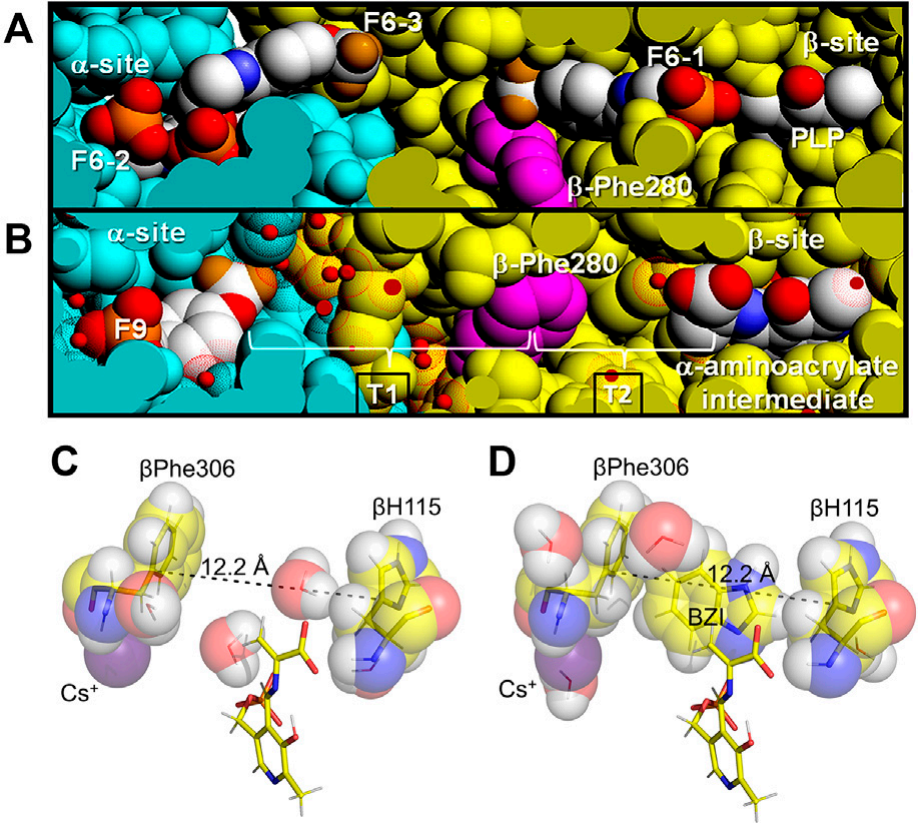
Tunnel views and Indole sub-site views at the VDW radii. **(A)** View showing F6 bound to the three sites identified in PDB ID: 4WX2 and designated as F6-1, F6-2, and F6-3 (CPK colors) compared with **(B)** the structure of the α-aminoacrylate intermediate (PDB ID: 4HN4). In these VDW views, some of the amino acid residues forming the channel have been cut away (slabbed) to expose the tunnel interior and reveal the locations of the three F6 molecules bound within the tunnel and the α-site. Water molecules are shown as small red balls inside dot surfaces at the VDW radius of oxygen. Color scheme: α-subunit residues, powder blue, β-subunit residues gold. The location of βPhe280 (magenta) is also shown. The brackets indicate the hydrophilic (T1) and hydrophobic (T2) regions of the tunnel. Figure redrawn from Hilario et al. (2016). **(C)** VDW contact structural detail of the β-subunit indole sub-site in E(A–A) (PDB ID: 4HN4) with hydrogens (white) modeled onto the heavy atoms. **(D)** E(A–A) (BZI) complex (PDB ID: 4HPX) showing structure detail of the indole subsite occupied by the indole isostere BZI including the VDW contacts of BZI with residues βPhe306 and βH115. The subsite distance of 12.2 Å is just right to bind BZI. The monovalent cation sites in these structures are occupied by Cs^+^. Coloring scheme: carbons, yellow and in VDW spheres overlapping the stick representations, while the Cs^+^ ions are colored purple.

Direct transfer of a substrate between the two catalytic sites of a bienzyme complex is the simplest enzyme example of channeling. TS was the first enzyme system demonstrated to channel a common intermediate (indole) in a bienzyme complex. (Hyde et al., 1988; Dunn et al., 1990; Lane and Kirschner, 1991). The intermolecular tunnel in TS extends from the α-catalytic site near the α-β subunit interface to the β-catalytic site, a distance of ∼30 Å (Hyde et al., 1988) (Figures 5A,B). This tunnel is comprised of two sections (T_1_ and T_2_, Figure 5B). The first section (T_1_) is a relatively hydrophilic region extending from the indole ring subsite of the α-subunit into the β-subunit to tunnel residues βTyr279 and βPhe280 (T_1_, Figures 5A,B) (Hilario et al., 2016). This section is filled with a hydrogen-bonded network of water molecules (Hilario et al., 2016). The second section (T_2_) is a very hydrophobic, dewetted nanotube and extends from βY279/βF280 into the indole ring sub-site of the β-catalytic site. There are no waters detected in this region of the tunnel (Hilario et al., 2016).

The exclusion of water from T_2_ and the E(A-A) site provides an essentially non-aqueous environment for the C-C bond forming step between the indole ring C_3_ and the α-aminoacrylate C_β_ carbons. While it previously has been speculated that βPhe280 plays a gating role in the channeling of indole (Anderson, et al., 1995), the findings of Hilario et al. (2016) indicate the transfer of indole is unhindered by βPhe280, a finding consistent with many of the x-ray structures of E(A-A). Hilario et al. (2016) examined the properties of the TS tunnel via x-ray crystallography, MD simulation and flexible docking studies. They reported the structures of complexes of TS with F6 bound to three different loci, one at the α-site and one within the β-subunit portion of the tunnel making contacts with βPhe280, and one bridging the α-β subunit interface (Figures 5A,B). The MD simulations indicated the following: 1) the hydrophobic region of the tunnel, T_2_, excludes water, consistent with a dewetted state which prevents the transfer of water between the α- and β-sites (the nonpolar portion of F6 binds to this region of the tunnel, another F6 molecule binds to the hydrophilic region, T_1_, of the tunnel, Figure 3), 2) in the E(A-A) intermediate the tunnel properties allow the transfer of indole from the α-site into the β-site even when βPhe280 partially restricts the cross-section of the tunnel (Figures 5A,B), 3) therefore, βPhe280 does not play a mechanistic gating role in the channeling of indole from the α-site to the β-site of E(A-A) during the β-reaction. These conclusions are further supported by rapid kinetic studies. The rate of reaction of indole with the α^T^β^R^ form of E(A-A) is very fast relative to the turnover rate of the β-rection, as is the rate of reaction of IGP with E(A-A) (Lane and Kirschner, 1983; Drewe and Dunn, 1986; Brzovic P. S. et al, 1992; Kulik et al., 2002). Since these reaction rates significantly exceed the turnover rate of the β-reaction, βPhe280 appears not to have a significant gating role. Therefore, the primary role played by the βPhe280 side chain is simply to contribute to the hydrophobic environs of the tunnel. Nevertheless, Brownian dynamics simulations using a residue-based coarse-grained model suggest that the channel does not always exist, and it may be blocked before TS reaches its final substrate bound conformation. This modeling work highlights the roles of protein conformations in substrate channeling (Fatmi and Chang 2010).

### The roles played by allosteric transitions in TS catalysis

As has been shown, the ligand-mediated allosteric interactions between the α- and β-subunits achieve the efficient utilization of IGP as the source of indole *via* synchronization of the α- and β-reactions (Dunn et al., 1987; Houben et al., 1989; Dunn et al., 1990; Houben and Dunn, 1990; Leja et al., 1995; Pan et al., 1997; Dunn et al., 2008; Dunn, 2012), the channeling of indole from the α-site to the β-site via the interconnecting tunnel (Hyde et al., 1988; Dunn et al., 1990; Kirschner, et al., 1991, Anderson et al., 1991; Brzović et al., 1992a, b; 1993), and the facilitation of chemical steps in the β-reaction (Ngo et al., 2007b). We hypothesize that the channeling of indole between the α- and β-subunits and the synchronization of the α- and β-reactions likely evolved to prevent the escape of indole and ensure the efficient utilization of IGP (Yanofsky and Rachmeler, 1958; Yanofsky and Crawford, 1972; Creighton, 1970); Miles, 1979; Pan et al., 1997; Miles et al., 1999; Yanofsky, 2007; Dunn et al., 2008; Dunn, 2012; Miles, 2001). These elements of the biosynthesis of L-Trp are important for organisms that utilize a Trp operon (Yanofsky, 2007).

### Monovalent cations are allosteric effectors of TS

The catalytic activity of the monovalent cation-free enzyme is strongly impaired (Peracchi et al., 1995; Woehl and Dunn, 1995a, b; Woehl and Dunn, 1999a, b; Mozzarelli et al., 2000; Weber-Ban et al., 2001). The activity of monovalent cation-free TS is decreased 45-fold compared to the Na^+^-activated enzyme. A wide variety of monovalent cations (MVCs) bound to the TS metal coordination site activate the α_2_β_2_ bienzyme complex, including Na^+^, K^+^, Cs^+^, NH_4_^+^ and guanidinium ion (**10**) (Fan et al., 1999; Mozzarelli et al., 2000; Dunn, 2012). The available structural information shows a binding site within the β-subunit that can accommodate Na^+^, K^+^, NH_4_^+^ or Cs^+^ (Rhee et al., 1996; Fan et al., 1999, 2000; Dierkers et al., 2009) (Figure 5C).

**FIGURE 6:**
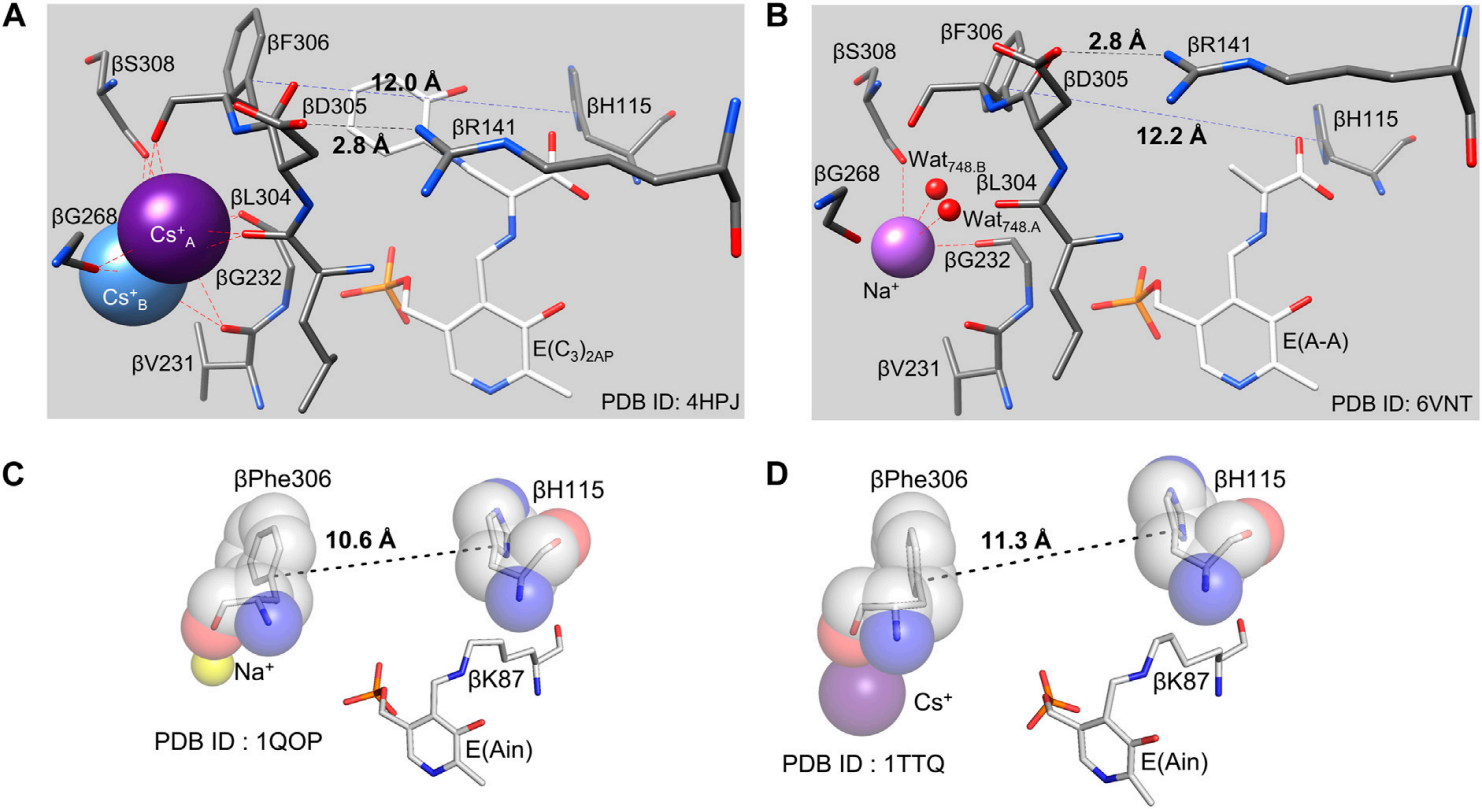
**(A,B)**, Comparisons of the pre-formed indole binding sites and the monovalent cation sites of the E(C_3_)_2AP_(Cs^+^) complex **(A)** and the E(A–A) (Na^+^) complex **(B)**. The distances spanning the indole sub-site cavity between the Cɣ atoms of βPhe306 and βHis115 (12.0 and 12.2 Å, respectively) are just right to match the VDW surface of indole (viz., Figure 5). In the E(Ain) monovalent cation complex with Na^+^ **(C)**, the subsite distance between βPhe306 and βH115 is 10.6 Å and is too small to accommodate indole. In the E(Ain)Cs^+^ complex **(D)**, the βPhe306 and βH115 distance is only slightly too small. (PDB IDs: 4HPJ, 6VNT, 1QOP and 1TTQ).

Kinetic studies have shown that monovalent cation binding is essential both for catalysis of the β-reaction and for the transmission of allosteric signaling between the β- and α-sites (Rhee et al., 1997; Woehl and Dunn, 1995a, b; Woehl and Dunn, 1999a, b; Mozzarelli et al., 2000; Weber-Ban et al., 2001; Fan et al., 1999, 2000; Dierkers et al., 2009). Interestingly, the Na^+^, K^+^, NH_4_^+^ forms of TS activate the reaction of L-Ser in the β-reaction by ∼30, ∼26, and ∼40-fold respectively (Weber-Ban et al., 2001), while the reaction of L-Ser with monovalent cation-free TS does not activate the α-reaction even though the monovalent cation-free enzyme forms an α-aminoacrylate species (Weber-Ban et al., 2001). The different monovalent cation-bound forms of TS give different distributions of intermediates along the β-reaction path with Na^+^ favoring E(Ain)^T^ and E(Aex_1_)^T^ and Cs^+^ favoring E(A-A)^R^ and E(C)^R^ (Weber-Ban et al., 2001; Dierkers et al., 2009). Inspection of the available x-ray crystal structures indicates that the variation in ionic radii of the monovalent cations causes a concomitant variation in coordination number and geometry (Woehl and Dunn, 1995b; Dierkers et al., 2009) which influences the dimensions of the β-subunit indole sub-site. The x-ray crystal structures of various monovalent cation-substituted TS enzymes identify the coordination site(s) as a cavity bounded by the backbone carbonyls of βVal231, βGly232, βGly268, βLeu304, βPhe306 and βSer308, residues that are not part of the COMM domain. Owing to the differences in ionic radius, Na^+^ only coordinates to three of the carbonyls (βGly232, βPhe306 and βSer308) and two waters, whereas Cs^+^ nearly fills this cavity and typically coordinates to 5 or 6 of the carbonyl oxygens). The K^+^ complex incorporates three of the carbonyl oxygens and a single water (Rhee et al., 1997; Dunn et al., 2008; Miles et al., 1999; Barends et al., 2008a, b; Ngo et al., 2007a b). Of special note is the involvement of βPhe306 in this cavity and the linkage of this residue to βAsp305. The x-ray structures show the side chain phenyl ring of βPhe306 is a component of the β-subunit indole sub-site while βAsp305 plays an important role in the stabilization of the R state conformation. Thus, the stabilizing effect exerted by Cs^+^ on the R state likely has its origins in the coordination of Cs^+^ by βPhe306 and the concomitant effect on the positioning of the βAsp305 side chain carboxylate in the conformation needed for formation of the salt bridge with βArg141 in the β-subunit R state conformation. This interaction also stabilizes the positioning of the βPhe306 phenyl group so that the VDW dimensions of the indole sub-site match the VDW dimensions of indole, creating a preformed sub-site (Figures 5C, 6A–D). Clearly, the movement of the COMM domain as the β-subunit switches from the T state to the R state causes the indole sub-site to expand from dimensions too small to accommodate the binding of indole (T state) to a dimension of 12.2 Å that matches the VDW surface of indole. Thus, the Na^+^ complex stabilizes the T-state (Dierkers et al., 2009) while Cs^+^ favors complexes with sub-sites that match or nearly match the dimensions of indole.

In 1996, Peracchi et al. (1996) reported that the pH dependent interconversion of E(Aex_1_) with E(A-A) is modulated by proton binding and involves two groups with apparent pKa values of ∼7.8 and ∼10.3. Since this pH dependence is not observed in the β_2_ dimer (Peracchi, et al., 1996), it most likely arises from the allosteric properties of α_2_β_2_. The microscopic origins of these ionizations are not known, but this behavior is reminiscent of the Bohr effect on dioxygen binding to hemoglobin (Signore et al., 2021). In TS, we speculate that these pH effects have origins in the ionizations of βLys87 and the salt bridging interactions linked to the T-to R-state transition (Peracchi et al., 1996; Phillips et al., 2005; 2008a, b).

**FIGURE 7:**
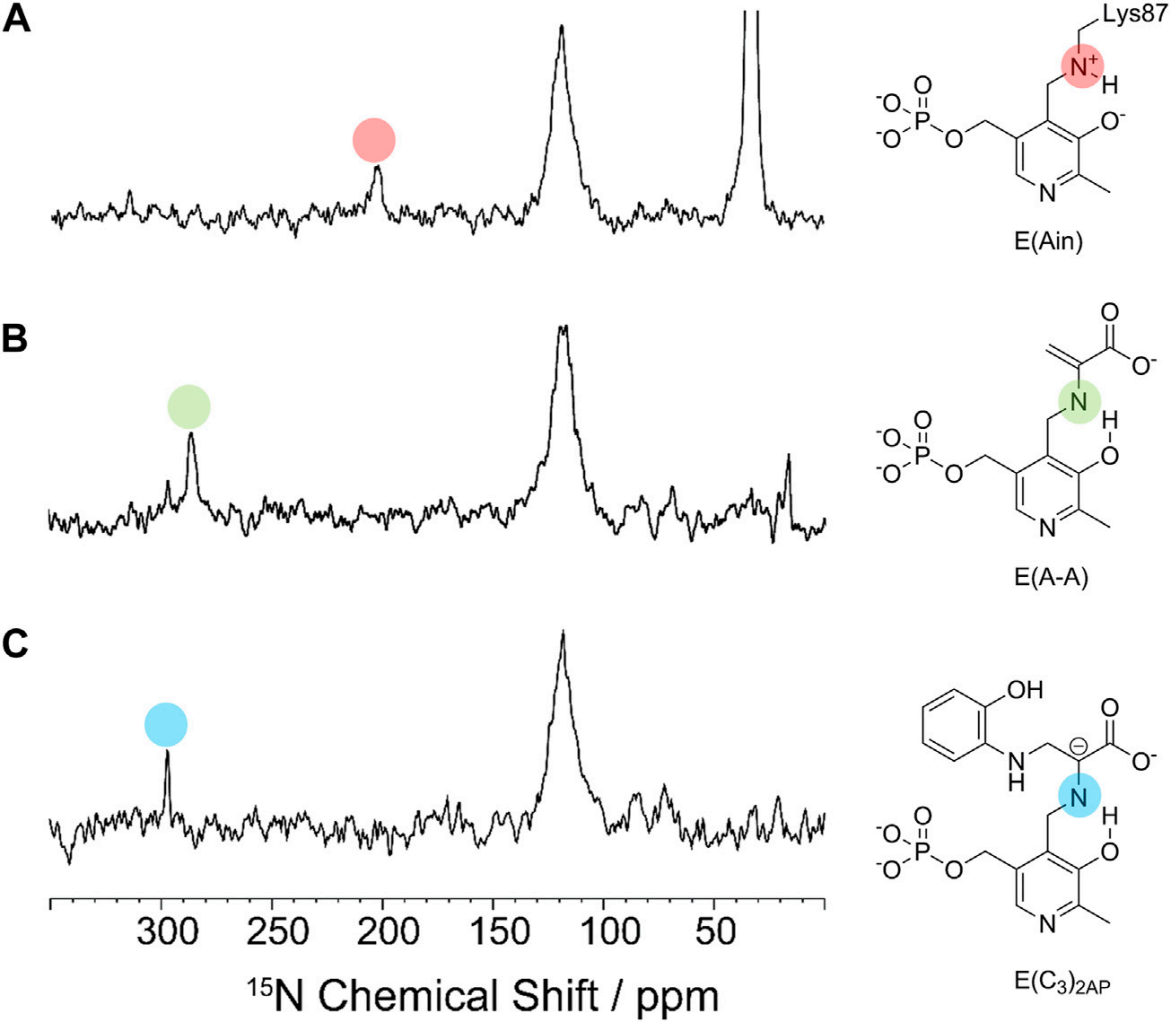
^15^N CPMAS spectra of **(A)** E(Ain), **(B)** E(A–A) and **(C)** E(C_3_)_2AP_. The indole analogue 2-aminophenol (2AP, **12**) reacts with E(A–A) to give a quasi-stable carbanion intermediate, E(C_3_)_2AP_, that turns over slowly to yield the corresponding new amino acid analogue of L-Trp (Ferrari et al., 2001). Spectra acquired at 9.4 T and 8 kHz magic-angle spinning (MAS) at −10°C. Resonances assigned to the Schiff base nitrogen are indicated by the red and green dots for the E^R^(A–A) and E^R^(C_3_)_2AP_ intermediates and fall at approximately 286.0 and 298.6 ppm, respectively (Caulkins et al., 2016; Holmes et al., 2022), indicating that the deprotonated Schiff base nitrogen is the dominant tautomer. For the E(Ain) intermediate, the Schiff base nitrogen resonance at 202.3 ppm (Caulkins et al., 2014) indicates that the protonated Schiff base nitrogen is the dominating tautomer.

The cofactor protonation states determined by solid-state NMR reflect the relative stabilities of the allosteric states observed in TS. For example, in the T state complex of E(Ain) the PLP moiety has a protonated Schiff base nitrogen (Figure 7A) (Caulkins et al., 2014; Klein et al., 2022), whereas the Schiff base nitrogen in the R state complexes of E(C_3_)_2AP_ and E(A-A) have deprotonated Schiff base nitrogen states as the dominating tautomers (Figures 7B,C) (Caulkins et al., 2016; Holmes et al., 2022). We postulate that this difference in protonation states has its origins in the presence of active site water molecules in the E(Ain)^T^ conformation while the E(A-A)^R^ and E(C_3_)^R^ conformations are significantly more dehydrated (Huang et al., 2016).

### The TS allosteric transitions: The α-subunit

**FIGURE 8:**
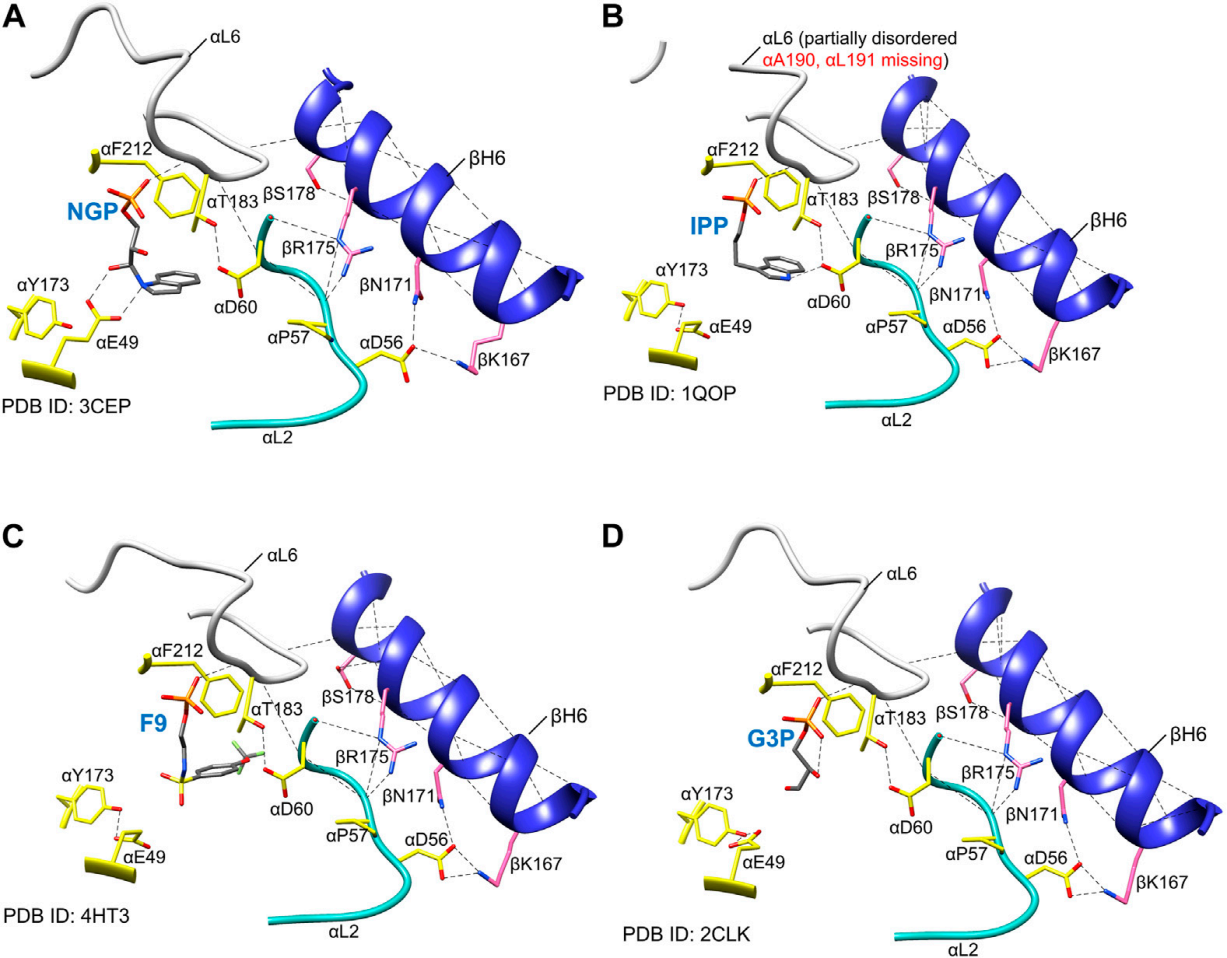
Examples of the positioning of α active site residues, αβ contacts and order in loop αL6 in the presence of different α site ligands. **(A)** NGP (**15**) (Kulik et al., 2005), **(B)** IPP (**13**) (Hyde et al., 1988), **(C)** F9 (**8**) Ngo et al., 2007a, **(B)**, **(D)** product G3P (**6**) (Ngo et al., 2007a). Notice that the αE49 side-chain points away from the α site ligands in each of the panels, whereas αD60 side-chain H-bonds to the aromatic nitrogen (N-1) atom of the IGP analog IPP, as depicted in Figures 2A, 6, it would play a central role in IGP cleavage. αF212 is rotated towards the ligand, providing a hydrophobic environment for the cleavage. Notice that NGP has been proposed to be a transition state analogue for the α-site catalyzed cleavage of IGP (Kulik et al., 2005). The essential hydrogen bonding interaction between αThr183 and αAsp60 is shown together with those between αL2, αL6, and βH6. Coloring scheme: protein side chains, yellow sticks; loop αL6, white ribbon; loop αL2, teal ribbon; helix βH6, blue ribbon. The α site ligands are shown as sticks in CPK colors; plausible H-bonds, black dashed lines. Image rendered in Chimera 1.15 (Pettersen et al., 2004).

The PDB archive (Berman et al., 2000) shows that TS undergoes conformational transitions that alter the structures of the α- and β-subunits, and that the α-subunit switches between an ensemble where loop αL6 is disordered in the ligand-free α-site (Hyde et al., 1988; Miles, 2001; Kulik et al., 2005; Ngo et al., 2007b; Yanofsky, 2007) and an ensemble wherein loop αL6 is well ordered when G3P, or substrate analogue F9 are bound (Kulik et al., 2005; Miles et al., 1999; 2001; Ngo et al., 2007a, b; Barends et al., 2008a, b; Lai et al., 2011; Niks et al., 2013; Caulkins et al., 2016; Ghosh et al., 2021) (Figures 2, 8). This disordered-to-ordered transition of αL6 also induces an ordering of loop αL2. Both loops make contributions to the α-β subunit interface via helix βH6 of the COMM domain (Miles et al., 1999, 2001; Kulik et al., 2005; Ngo et al., 2007b; Barends et al., 2008a).

Structural and kinetic evidence indicates that the disordered αL6 loop renders the α-subunit essentially catalytically inactive (Brzovic et al., 1992a; 1993; Kulik et al., 2005; Miles et al., 1999; 2001). The NMR experiments of Sakhrani et al. (2020) on the solution structure of the α-subunit free and bound to the IGP analogue F9 show similar effects on the affinity of ligands for the α-subunit (∼20-fold in α^T^β^T^ and ∼500-fold in α^T^β^R^). When the α subunit is switched to the closed conformation (R state), loop αL6 (residues α179–α193) folds down over the catalytic site and makes interlocking interactions with loop αL2 (residues α56–α60) and helix βH6, thus creating a local environment where the ligand is shielded from solution, and access from solution into the interconnecting tunnel is blocked, Figures 2, 8A (Miles 2001; Harris and Dunn, 2002; Harris et al., 2005; Kulik et al., 2005). Within these complexes, αThr183 swings into a position where the side chain hydroxyl forms a hydrogen bond to one oxygen of the αAsp60 carboxylate, stabilizing this catalytic group in the proposed position for α-site catalysis Kulik et al., 2002) (Figure 8). Notice in Figures 1B, 8A, the conformation of the αGlu49 side chain carboxylate group also becomes stabilized in the position necessary to act as an acid-base catalyst in facilitation of the cleavage of the C-C bond. So long as the α-subunit retains this conformation, indole formed via scission of IGP is prevented from dissociation into solution, and instead, is shuttled into the tunnel leading to the β-subunit catalytic site 25 Å away. Consequently, the allosteric transition of the α-subunit occurs via a transition of loop αL6 between disordered and ordered states and induces a reordering of αL2 (Miles, 2001; Kulik et al., 2002; Barends et al., 2008a; Raboni et al., 2009; Dunn, 2012; Maria-Solano et al., 2019; Teixeira et al., 2020) (Figure 8). The formation of well-ordered αL6 and αL2 loops creates a well-defined subunit interface between the α- and β-subunits, an α-site with high affinity for IGP, and configures αAsp60 for catalysis (Figure 8). While the x-ray structural evidence clearly supports the involvement of two subunit conformations, T and R, and four quaternary states, α^T^β^T^, α^R^β^T^, α^T^β^R^ and α^R^β^R^ (Niks et al., 2013; Ghosh et al., 2021), it is also clear that the binding of G3P or analog GP can give complexes where loop αL6 is only partially disordered and the extent of disorder depends, at least in part on the structure of the ASL. For example, Ngo et al. (2007b) reported evidence for three conformational states, open, partially closed, and closed of the α-subunit. They found that in the absence of α-site substrates or substrate analogues both the α- and β-sites reside in open conformations and αL6 is completely disordered. In structures where the β-domain is closed and the α-site is occupied by an ASL, the electron densities for loop αL6 (residues α179-193) are well-defined (with the exception of the two outermost residues in some structures). These structures clearly exhibit closed α- and β-subunits. In x-ray crystal structures of IGP bound to the α site of the internal aldimine (PDB ID: 2RH9, 2RHG, 1QOQ), the α subunit adopts a T conformation with a disordered αL6 loop and suggest that these IGP complexes represent an inactive state of the α subunit. In structures where the β-subunit is not closed and the α-site is occupied, typically parts of αL6 are visible in the electron density maps and may represent a partially closed state. What is now clear is that the partial disorder observed in some αL6 structures likely is a consequence of structural mismatching between the ASL and the α-subunit (vis. IPP). Since IPP lacks hydroxyls at the 2 and 3 carbons, the hydrogen bonding between site and substrate is not replicated in the IPP complex, Figure 8B. The complexes with the IGP analogue, F9, and the proposed transition state analogues 1-(2′-hydroxyphenylamino) 3-glycerolphosphate (2-HGP, **14**) and N-(indolinyl) 3-glycerolphosphate (NGP, **15**) (Figures 4, 8) provide sufficient interactions with αL6 to stabilize the interaction between all of the residues in αL6 and the surface of the α-subunit (Kulik et al., 2002, 2005; Dunn 2012).

It is interesting to note that the x-ray structure of the α_2_β_2_ TS complex found in *Pyrococcus furiosus* TS shows the α-subunits with the closed conformation and a well-ordered αL6 loop (Lee et al., 2005). Nevertheless, this homolog of the Escherichia *coli* and *Salmonella enterica* serovar *Typhimurium* enzymes shows many of the allosteric properties exhibited by the enteric tryptophan synthases.

### The TS allosteric transitions: The β-subunit

**FIGURE 9:**
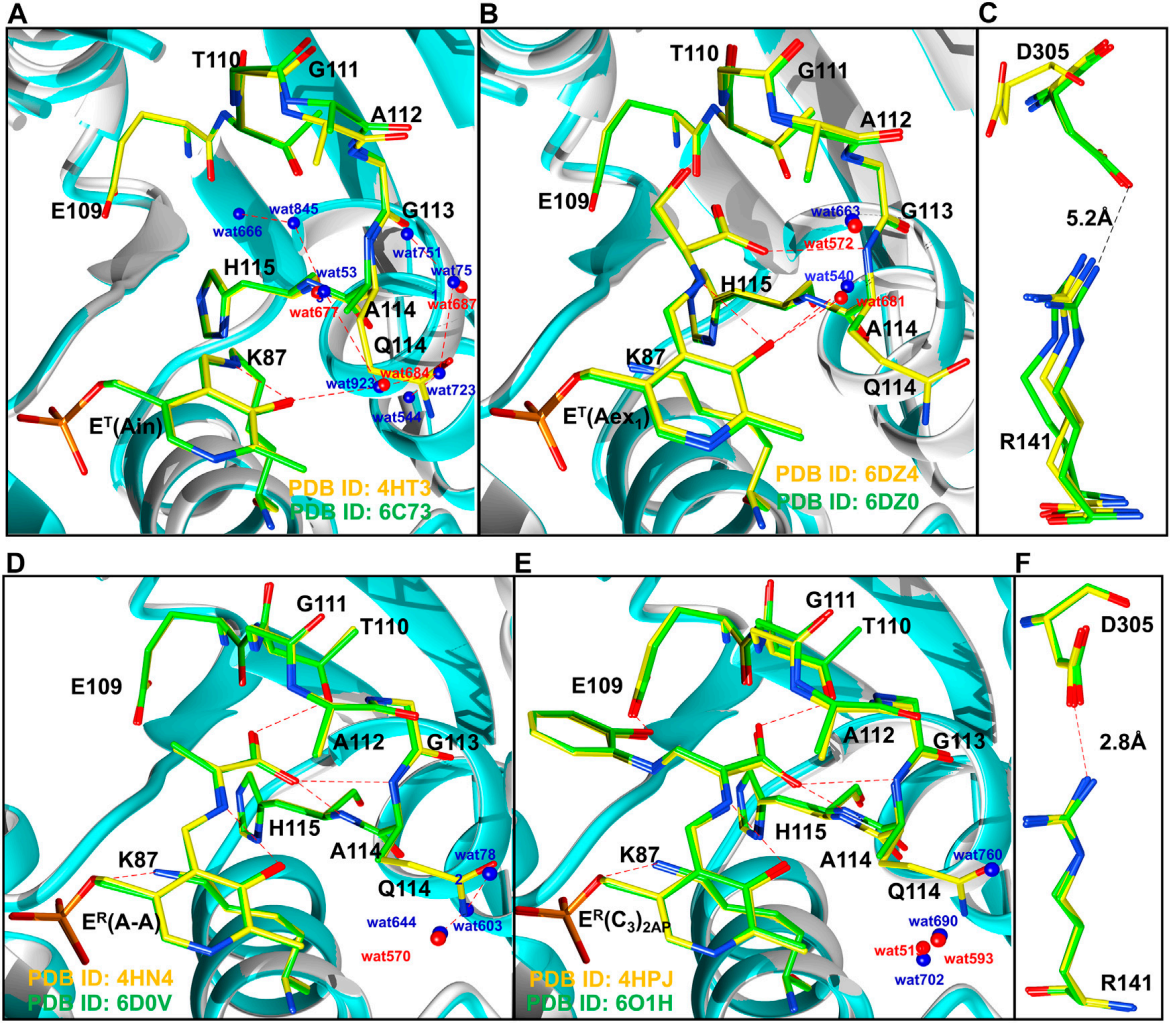
Comparison of T state **(A,B)**, and R state **(D,E)** conformations of TS intermediates. **(A)** wild-type (PDB ID: 4HT3, 6DZ4) and βQ114A (PDB ID: 6C73, 6DZO) internal aldimine and **(B)** external aldimine complexes. Coloring scheme: wild-type, yellow carbons and red waters; βQ114A, green carbons and blue waters. Wild-type ribbons gray, βQ114A ribbons, teal. **(A)**: Comparisons of wild-type and βQ114A internal aldimines. **(B)**: Comparisons of wild-type and βQ114A TS external aldimines. **(C)**: The βR141 and βD305 residues in the T state (open) complex of the β subunit are too distant to form an H-bonded salt bridge in E(Ain and E(Aex_1_) complexes. **(D)** Comparison of wild-type and βQ114A E(A–A). **(E)** Comparison of wild-type and βQ114A E(C_3_)_2AP_. **(F)** βR141-βD305 salt bridges found in E(A–A) and E(C_3_)_2AP_. Figure redrawn from Ghosh et al. (2021).

In contrast to the α-subunit, the β-subunit allosteric transition involves a relatively modest conformational change wherein an 88 amino acid residue domain (the COMM domain, residues β102—189) moves as a rigid body, undergoing a slight rotation and translation. This motion causes a displacement of COMM domain residues by ∼ 2.6 Å along the αβ subunit interface and by ∼ 4.5 Å near the β-catalytic site (Figure 2). While this motion is relatively modest, the functional changes are large. The translation and rotation of the COMM domain causes the β-subunit to switch between two states, one open (the T state) the other closed (the R state) (Figure 2). The COMM domain contributes key residues and structural units essential both to catalysis and to allosteric communication (Figures 2, 3). One surface of the COMM domain forms a wall of the cleft linking the β-site and solution. Residues from the COMM domain and from loop αL2 provide most of the contacts that comprise the α-β subunit interface (Figures 2, 8). The COMM domain also contributes the following components of the allosteric response: the substrate carboxylate recognition sub-site (loop βL3) (Figures 2, 8) and forms part of the indole recognition sub-site (Figures 5, 6); a catalytic site residue, βGlu109, and a salt bridge forming residue, βArg141, forms a strong Coulombic interaction with βAsp305 that stabilizes the β^R^ conformation (Ferrari et al., 2001, 2003) (Figures 6, 9). In the transition of α^T^β^T^ to α^R^β^R^, the combined motions of the COMM domain and loops αL6 and αL2 close the entrance into the α-site and tunnel from solution (Figure 2) and the movement of the COMM domain also closes the cleft into the β-site from solution (Figure 2) (Harris and Dunn, 2002; Harris, et al., 2005). Thus, the switch to the α^R^β^R^ state generates steric constraints (among the ensemble of closed conformation states) that prevent the escape of indole from the enzyme α-site and the tunnel, creates the indole sub-site at the β-catalytic site, and blocks the transfer of L-Ser and L-Trp between the enzyme β-site and solution.

The β^T^ catalytic site resides in a solvent-exposed environment where three water molecules solvate one face of the PLP moiety and a lattice of waters extends to the aqueous milieu (Ghosh et al., 2021). In these complexes the β-site is accessible to bulk solvent via the narrow cleft, allowing L-Ser and product L-Trp to enter and exit the β-catalytic site. The switch of β^T^ to β^R^ closes the cleft to a narrow aperture (∼3Å diameter, Figure 2) and only a single water molecule is retained on this face of the PLP moiety, creating a more hydrophobic environment (Ghosh et al., 2021). The aperture of the portal is too small to allow the entry/exit of small molecules such as indole, L-Ser or L-Trp. Thus, the small motion of the COMM domain triggers activation of IGP cleavage at the α-site, facilitates channeling of indole from the α-site to the β-site, creates the indole subsite, orchestrates the interconversions of E(Aex_1_), E(C_1_), E(A-A), E(C_2_), E(C_3_) and E(Aex_2_), synchronizes the catalytic cycles of the α- and β-reactions, and prevents the escape of indole from the confines of the α and β sites and the tunnel during the αβ catalytic cycle (Dunn, 2012; Hilario et al., 2016).

The efficient synthesis of L-Trp by TS requires the facile interconversion of T and R states during the overall αβ-rection. This is accomplished by constraining indole to the interior of the complex while allowing the ingress and exit of substrates at the α- and β-sites as needed. This balance requires that the energies of the T and R states modulate the binding of substrates and chemical intermediates so that the switching between T and R states is orchestrated with the appropriate chemical steps. Thus, binding of IGP and G3P to the α-site and formation of the α-aminoacrylate at the β-site drive the switching of α^T^ to α^R^ and β^T^ to β^R^.

**FIGURE 10:**
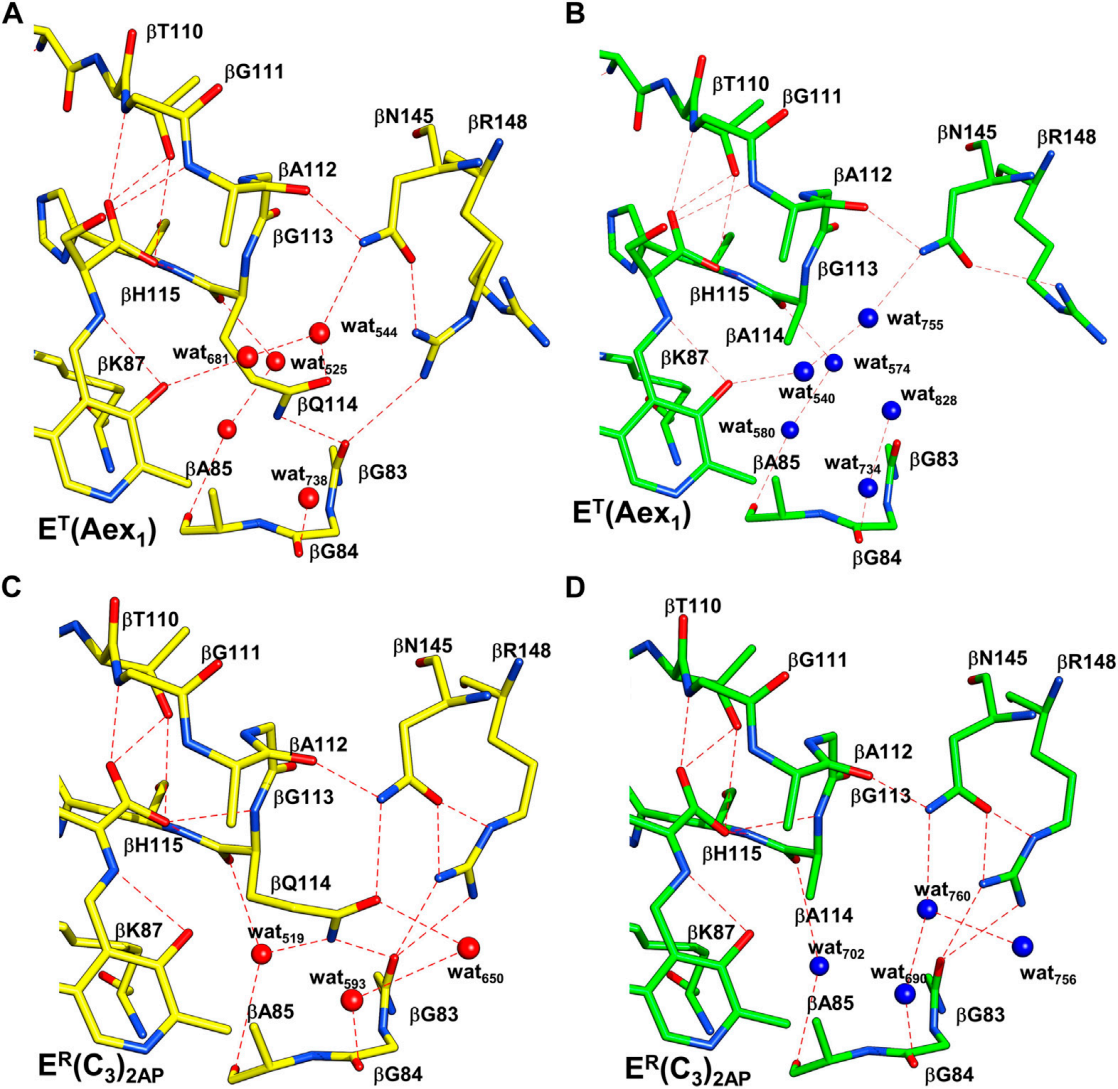
Comparison of the x-ray structures of T and R β-site conformations. Coloring scheme: WT structures, carbons yellow and waters red; βQ114A structures, Carbons green and waters blue. H-bonding interactions are shown as red dashes. **(A,B)**, WT and βQ114A E^T^(Aex_1_) complexes (PDB ID: 6DZ4, 6DZO, respectively). **(C,D)**, WT and βQ114A E^R^(C_3_)_2AP_ complexes (PDB IDs: 4HPJ and 6O1H). The Q to A mutation in T complexes **(A,B)** causes minor disruptions of the H-bonding network with waters and the neighboring protein residues. The R complexes **(C,D)** show larger differences, the H-bonding interactions among βQ114, βN145 and βR148 are lost. These changes are virtually the same in the closed structures of the E^R^ **(A–A)** complexes (not shown). Figure redrawn from Ghosh et al. (2021).

**FIGURE 11:**
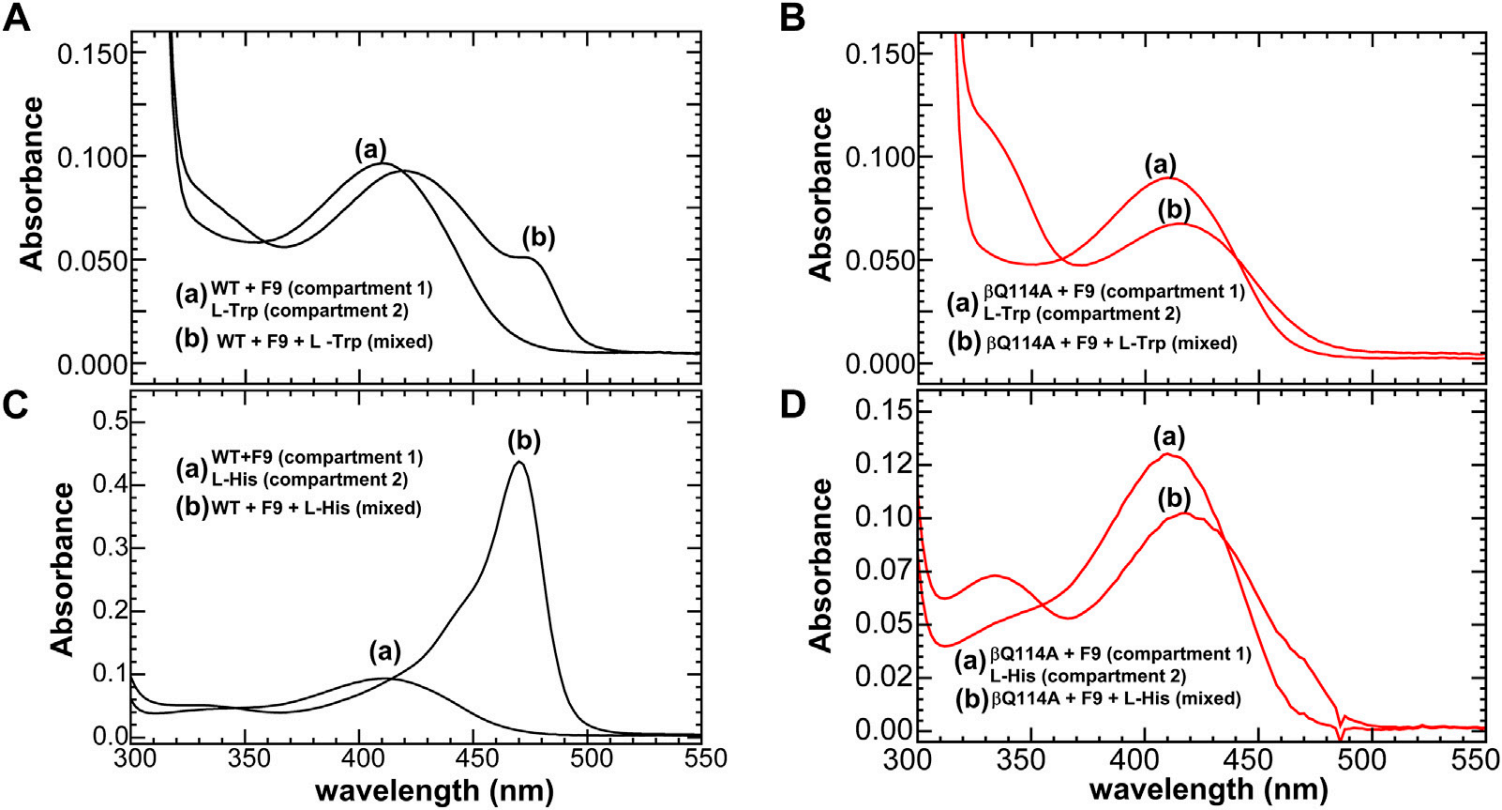
Reactions of WT and βGln114Ala TS, respectively with L-Trp **(A,B)**, and with L-His (**16**) **(C,D)**. A double-difference quartz cuvette was used to acquire the spectra. In each panel, the solutions in the two compartments of the cuvette are designated as follows: **(A)** spectra before mixing and **(B)** spectra following mixing. Color code: black WT, and red βGln114A. All the reactions were carried out in the presence of α site ligand F9. Figure redrawn from Ghosh et al. (2021).

These constraints also dictate that the relative energies of the T and R conformation states be sensitive to weak intramolecular interactions within loops αL2 and αL6 of the α-subunit and within the COMM domain of the β-subunit. For example, Ghosh et al. (2021) have demonstrated that the replacement of βGln114 by Ala interferes with the catalytic activity of the β-subunit by altering the relative stabilities of intermediates in the β-reaction. The origins of the altered stabilities appear to arise from the incorporation of water molecules into the cavity by replacement of the Gln side chain with the smaller Ala side chain, and by the loss of hydrogen bonding interactions between the side chains of βGln114 with βAsn145 and βArg148 in β^R^ (Figure 10). Since there are no intramolecular hydrogen bonds to the βGln114 side chain in the T state, the Gln to Ala mutation destabilizes the R state. This destabilization of the R state is illustrated by the change in distribution of intermediates resulting from reaction of L-His with TS (Figure 11). Mutation of either βArg141 or βAsp305 to Ala similarly alters the activity of the β-site and alters the distribution of aldimine and carbanion intermediates (Ferrari, et al., 2001, 2003). It previously has been argued that these mutations destabilize the R state by destroying the βArg141—βAsp305 salt bridge (Ferrari et al., 2001, 2003; Ghosh et al., 2021).

In wild-type TS, the interconversion of the E(Aex_1_) and E(A-A) species is accompanied by a switch from the T state to the R state. Early work (Rhee et al., 1997) demonstrated that mutation of the β-site catalytic residue βLys87 to Thr shifts the stabilities of the E(Aex_1_) and E(Aex_2_) intermediates strongly in favor of structures wherein both the α- and the β-subunit reside in closed conformations, i.e., α^R^β^R^. Thus, Rhee et al. (1997) determined the first two x-ray structures of the completely closed α^R^β^R^ state. Studies by Ferrari et al. (2001, 2003) have established that the destruction of the βD305-βR141 salt bridge (Figures 9C,F) by mutation of either of these residues to Ala alters the thermodynamics of the conformational transitions of the β subunit to the R state. This alteration results in the destabilization of E^R^(A-A), thus shifting the distribution of β reaction intermediates in favor of E^T^(Aex_1_) and an altered substrate specificity. Both the βAsp305Ala and βArg141Ala mutants give distributions of intermediates that are shifted in the favor of E^T^(Aex_1_). Recent studies (Ghosh et al., 2021) have shown that a mutation distal from the αβ interface such as replacing the βL3 residue βGln114 with an alanine have significant effects on the distribution of species in the β reaction. They have shown that in the reactions of L-His and L-Trp, ASL binding shifts the β subunit population mostly to the E^R^(C) species, while, with the βGln114A mutant, these reactions are significantly impaired and ASL binding shifts the population from the E^R^(C) species to a mixture of the E^T^(Aex) and E^T^(GD) complexes (Figure 11).

Using the standalone β subunit from *P furiosus* TS, Buller et al. (2015) were able to select for mutations that enhance the activity of the relatively inactive β-subunit to a level exceeding that of the wild-type complex. These findings suggest that the heterotropic allosteric activation achieved by formation of the αβ dimeric unit in the wild-type system can be mimicked by mutations that facilitate catalysis.

**FIGURE 12:**
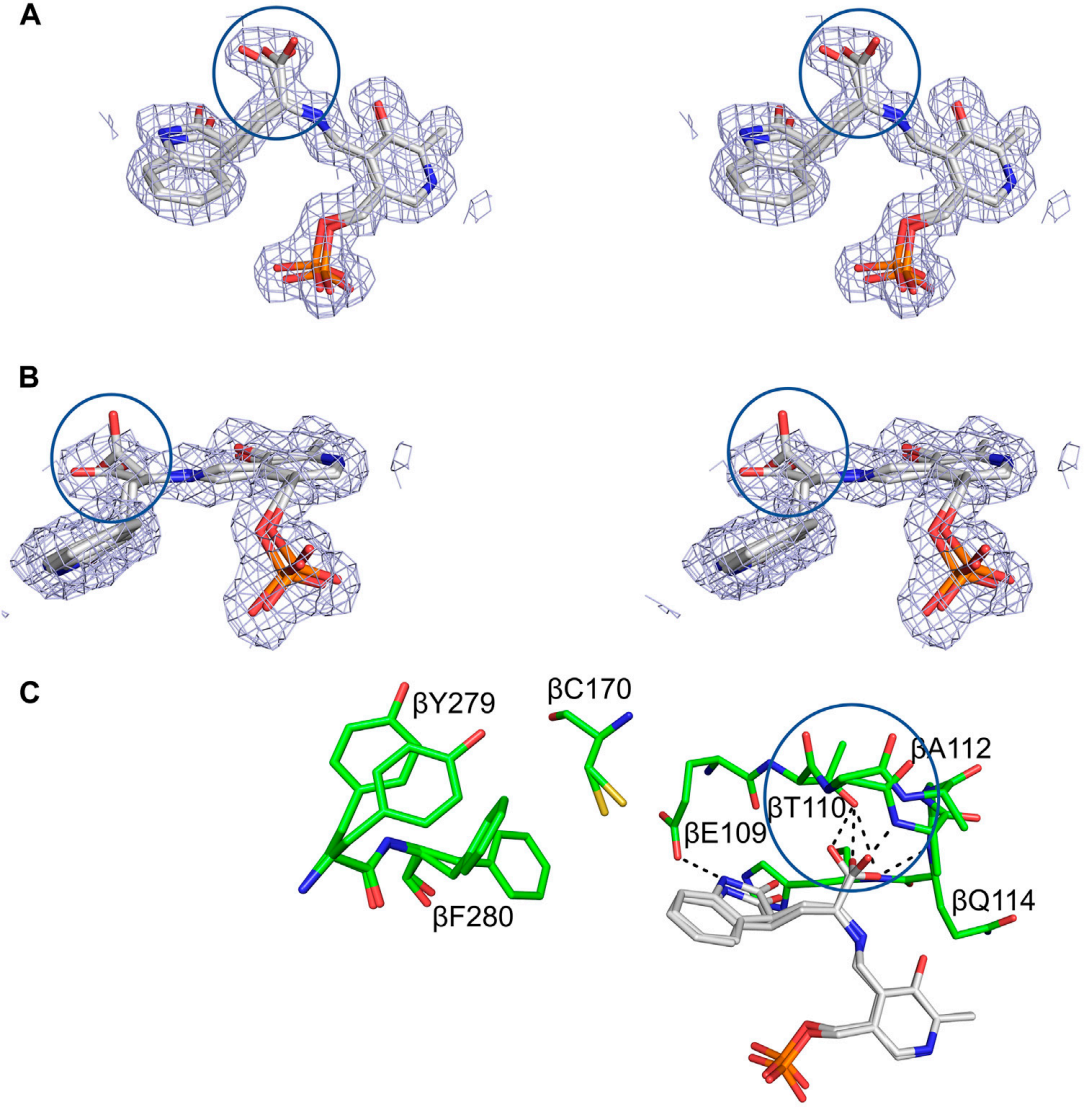
Structural detail of the TS β-site complexed with OIA. **(A,B)** Crossed-eye stereo views of the 2(Fo-Fc) electron density map at 1σ of the complex viewed from the side and top. **(C)** Stick representation of the interactions between OIA covalently bound to PLP in the OIA-PLP complex. Hydrogen bonding contacts of the OIA-PLP complex with the carboxylate binding loop are shown as black dashes. The complex contains both an external aldimine, with the α-carboxylate perpendicular to the PLP plane, and a carbanion structure, with the α-carboxylate in the PLP plane (circles). Loop βL3, β109-β114, adopts two different conformations to accommodate the carboxylates of the aldimine and carbanion complexes. Coloring schemes: **(A,B)** carbons white. **(C)** protein residues, carbons green; OIA, carbons white. All other atoms in standard CPK colors. Figure redrawn from Phillips and Harris (2021).

The complexes formed by the sodium form of TS in the reactions of L-Trp and D-Trp (**17**) (Lane and Kirschner, 1981; Drewe et al., 1989) and analogues of the carbanion intermediates formed with OIA (**18**) (Roy et al., 1988b) and DOA (**19**) (Phillips and Harris 2021) recently have been examined by UV/Vis spectroscopy and rapid kinetics, and the structures of these complexes with GP bound to the α-site have been solved by x-ray crystallography (viz., Figure 12) (Phillips and Harris 2021). These studies establish the stereochemistry of the OIA and DOA reactions and by analogy indicate the course of the stereochemical transformations during formation of E(C_2_) and E(C_3_) in the β-reaction. The x-ray structures also provide additional information about the allosteric states that predominate along the β-reaction pathway. The complexes of OIA and DOA give mixtures of both the external aldimine and the carbanion species within the same crystal while the subunit conformation is α^R^β^R^ (Figure 12). L-Trp gives a non-covalently bound complex (Buller et al., 2015) with the α^R^β^T^ conformation (Phillips and Harris 2021) whereas D-Trp gives a mixture of two external aldimine complexes with different orientations of the α-carboxylate, and a subunit conformation that also is α^R^β^R^. However, the β-subunit of one form of the heterodimeric unit in these mixtures fails to form the signature R-state salt bridge between βArg141 and βAsp305. These structures appear to model the interactions between the catalytic site and the reacting substrate during the interconversion of the external aldimine and carbanion intermediates (Figure 1C).

### Synchronization of the α- and β-reactions

We hypothesize that owing to the presence of a significant pool of L-Ser *in vivo*, the TS β-site exists within the bacterial cell primarily as the quasi-stable α-aminoacrylate species in the α^T^β^R^ conformation. In the absence of IGP, the α-site has the open α^T^ conformation, thus giving a TS resting state, α^T^β^R^, where the β-site is activated but sequestered away from small molecule nucleophiles that could cause deleterious side reactions (viz., Figure 3) (Blumenstein et al., 2007; Flynn and Downs, 2013; Hilario et al., 2016; Ghosh et al., 2021). This resting state appears primed for the binding and reaction of IGP at the α-site.

Because the conversion of E(Aex_1_) via E(C_1_) to E(A-A) at the β-site triggers activation of the α-site ∼30-fold (Anderson, et al., 1991; Brzovic P. S. et al., 1992), the synthesis of L-Trp via the α- and β-reactions begins when IGP binds and reacts at the α-site with the E(A-A) form of TS. The α-site remains in the α^R^ conformation until β^R^ is switched back to β^T^ when E(C_3_) is converted to E(Aex_2_) (Brzovic P. S. et al., 1992; Leja et al., 1995). Notice that the efficient transfer of indole from the α-site to the β-site *via* the interconnecting 25 Å tunnel requires that transfer occur within the α^R^β^R^. This closed conformation prevents the escape of indole.

**FIGURE 13:**
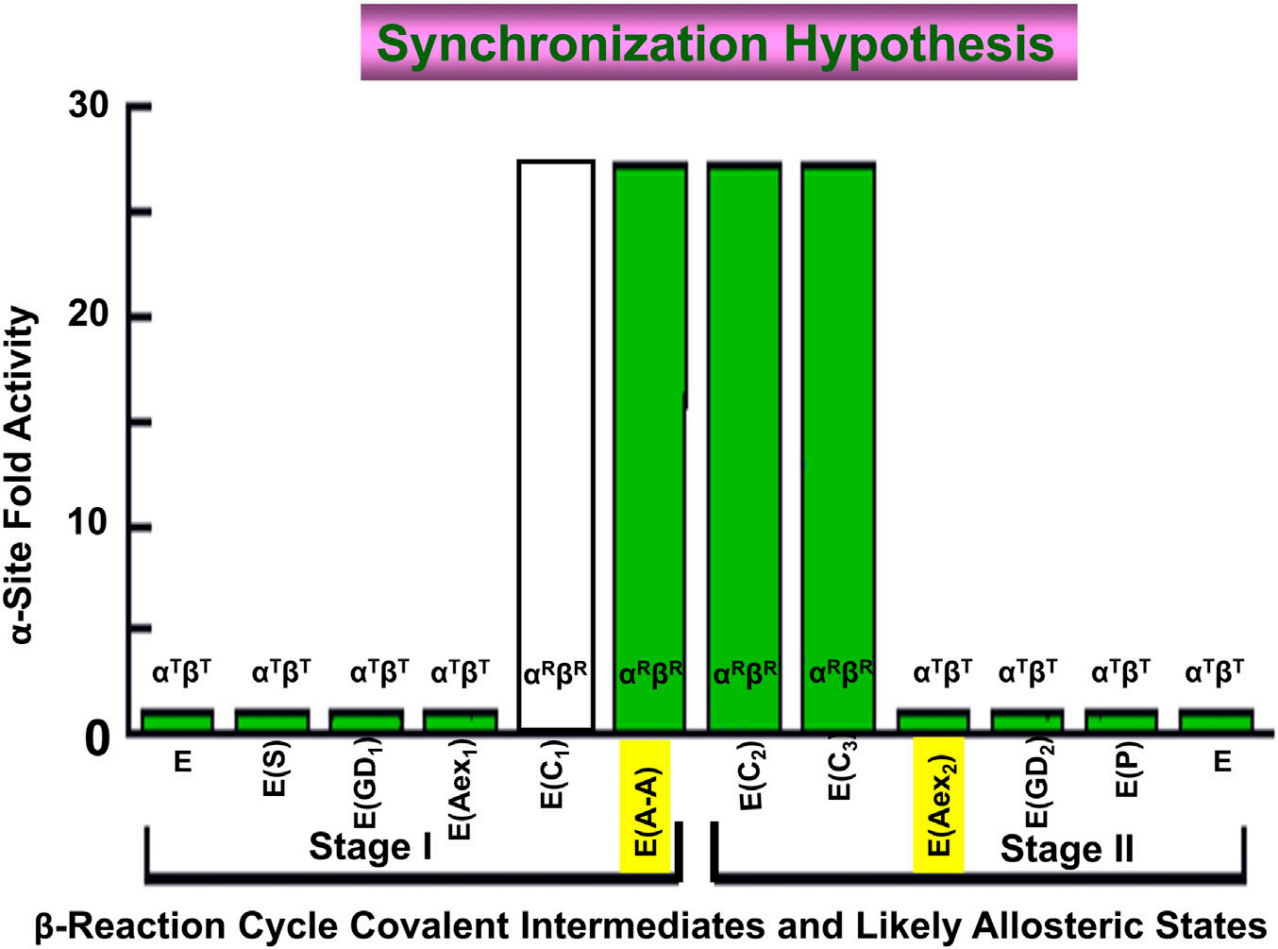
Graph depicting the dependence of the steady-state rate of the α-reaction (Y axis) on the covalent state of the β-site (X-axis). When E(Aex_1_) is converted to E(A-A) the α-site is activated >28-fold (Brzovic P. S. et al., 1992). The α-site is switched off again when E(C_3_) is converted to E(Aex_2_). This switching on and off ensures that the cycles of the α- and β-reactions are synchronized during the synthesis of L-Trp. Because the L-Ser carbanion is a fleeting species and there is no good analogue for E(C_1_) it is not known whether the activity switch occurs when E(Aex_1_) is converted to E(C_1_) or when E(C_1_) is converted to E(A-A). However, the experiments of Leja et al. (1995) are consistent with activation triggered by the conversion of E(Aex_1_) to E(C_1_). Figure redrawn from Brzovic P. S. et al. (1992).

One consequence of the switching between low and high activity states of the α-site in response to the switching of the β-subunit between β^T^ and β^R^ is to cause an in-phase synchronization of the α- and β-reactions (Figure 13) (Brzovic P. S. et al., 1992; Leja et al., 1995). This synchronization of the α- and β-reactions achieves the efficient utilization of the indole moiety of IGP for the biosynthesis of L-Trp by linking indole production *via* IGP cleavage at the α-site to L-Trp formation at the β-site. This linkage makes possible the channeling of indole between the α- and β-sites (Figure 13).

The relative stabilities of β^T^ and β^R^ are greatly affected by the binding and reaction of substrates to the catalytic sites of the αβ dimeric unit (Harris and Dunn, 2002, 2005). Dunn et al. (1990) reported clear evidence that the binding of the IGP analogue glycerol phosphate to the α-site strongly inhibits the reactions of indole and indole analogues with E(A-A) while the reactions with L-Ser or L-Trp are only slightly perturbed. These observations led to the conclusion that ASL binding switches the α-subunit to the closed conformation (the R state) preventing the entry of indole and indole analogues into the β-site via the α-site and the tunnel, while entry of L-Ser and L-Trp is unaffected, a conclusion in agreement with an α^R^β^T^ structure (Dunn et al., 1990) (Figure 2). These conclusions have been confirmed and expanded in more recent work (Brzović et al., 1992a, b; 1993; Harris and Dunn, 2002, 2005; Leja et al., 1995; Ngo et al., 2007b). One interesting and not fully explained feature of this inhibition is the observation that although high affinity ligands strongly inhibit the reactions of nucleophiles with E(A-A), all give residual reaction rates that become independent of the concentration of the α-site ligand at high ligand concentrations. Based on what we now know about the T to R allosteric transitions of TS, it seems likely that these residual reaction rates have their origins in the switching of E(A-A) complexes between β^R^ where entry and egress of small molecules is sterically blocked and β^T^ where small molecules are free to exchange between solvent and site. Thus, the slow residual rates likely are due to the rate of switching between β^R^ and β^T^and the rate of this switching is strongly influenced by the affinity of the ASL for the α-site.

## Discussion

### The allosteric regulation of tryptophan synthase

When taken together, the discoveries and advances described in the forgoing paragraphs combine to form a collage that captures at a structural and physio-chemical level the mechanism for the allosteric regulation of the tryptophan synthase bienzyme complex in enteric bacteria. The documentation in this collage shows tryptophan synthase allostery has become an important paradigm which begins to rival the hemoglobin allostery paradigm in terms of insight into the interplay between structure and function. However, the allosteric properties of tryptophan synthase and hemoglobin are very different, reflecting their very different biological functions. Allosteric interactions in TS are restricted to the regulation of substrate channeling in αβ dimeric units of the α_2_β_2_ complex that function to synthesize L-Trp from IGP, indole and L-Ser *via* an exquisite interplay of allosteric signaling that switches the αβ- and β-subunits between states of low and high reactivity. The allosteric transition of the TS α-subunit switches loop αL6 between disordered and ordered states, while the β-subunit transition causes a small motion of an 88 amino acid domain (Figure 2). Together, these motions synchronize the final two steps in the biosynthesis of L-Trp by reinforcing the correct placement and alignment of catalytic residues within an extended solvent-protected cavity that spans two active sites and an interconnecting, 25 Å-long tunnel (Figure 5). In contrast to TS, the allosteric properties of hemoglobin comprise a highly nuanced system of structure-function relationships that regulate the transport of dioxygen and nitric oxide by red blood cells into the tissues of higher organisms and the removal of CO_2_ and H^+^.

**FIGURE 14:**
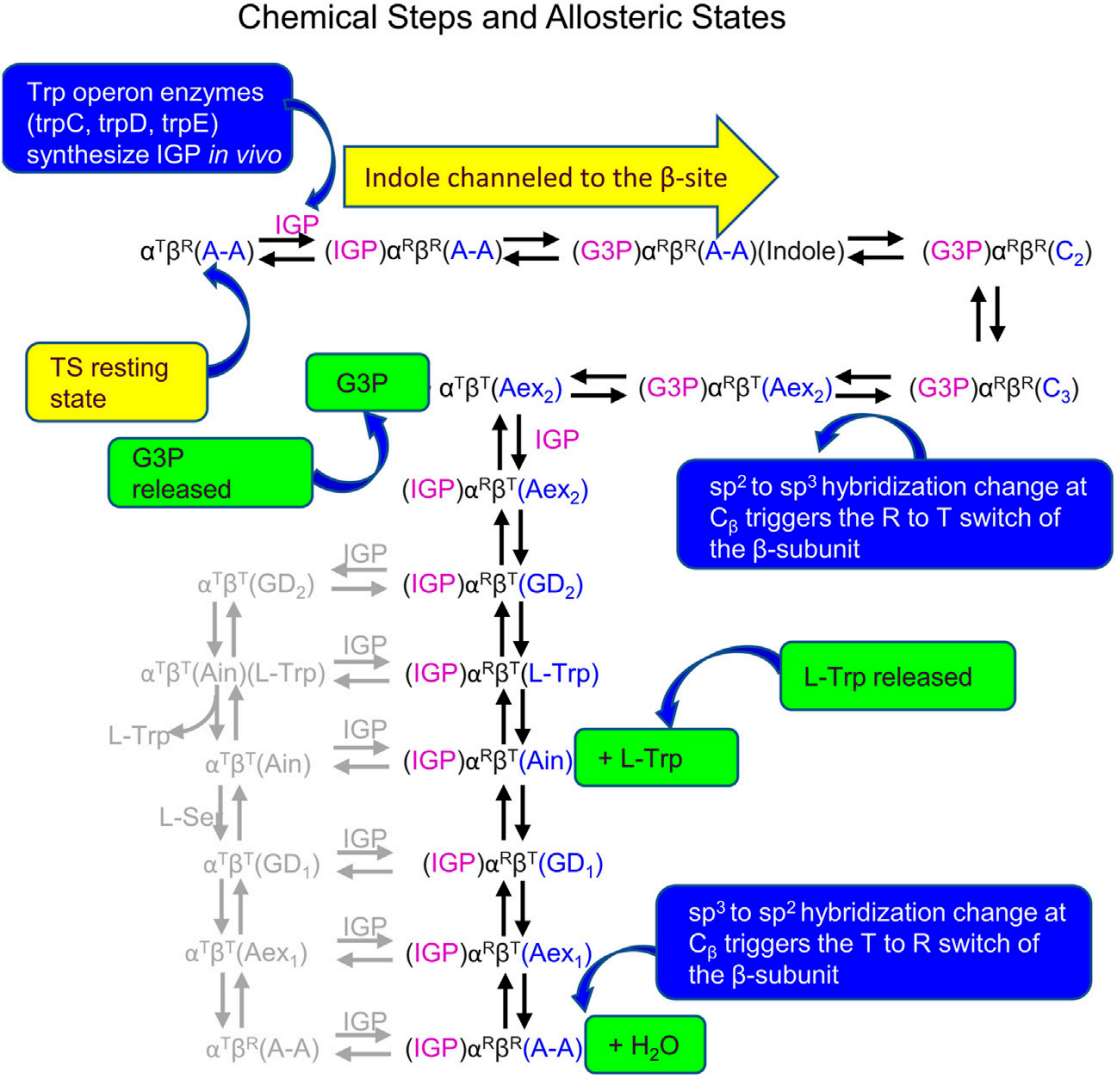
Summary of Chemical states and allosteric states proposed for the allosteric regulation of L-Trp synthesis *in vivo* by TS. Owing to the similarities of the energies of the T and R states, several of the chemical intermediates likely have comparable energies. Minor species thought to be present are shown in gray. The binding and release of substrates and products occur *via* the T state subunit conformations.

The reaction scheme presented in Figure 14 summarizes our current hypothesis for the allosteric regulation of L-Trp synthesis within the TS αβ-reaction cycle. This superposing links the known protein conformational states to the established chemical transformations that occur during a single round of catalysis. The presence of an L-Ser pool *in vivo* likely insures the αβ-reaction begins with the β-subunits of α_2_β_2_ in the form of E(A-A) and the allosteric units predominately α^T^β^R^ (Figures 2, 14). When IGP enters the open α^T^-site from solution, binding of IGP switches the α-subunit to the activated α^R^ state, and IGP is cleaved to G3P and indole. With TS in the α^R^β^R^ state, G3P remains bound to the α-site and indole is trapped within the confines of the α-site, the interconnecting tunnel, and the β-site. Because the tunnel functions as a selective filter that accommodates the passage of indole but rejects water and other polar molecules (viz. Figure 5), G3P remains bound to the α-site while indole is transferred via the tunnel into the indole subsite of the β-subunit as depicted in Figure 14 (Hilario et al., 2016; Ghosh et al., 2021).

Carbon-carbon bond formation at the β-site then occurs *via* nucleophilic attack of indole at C_β_ of E(A-A) giving E(C_2_) which is quickly converted to E(C_3_) while the allosteric unit remains in the completely closed state, α^R^β^R^ (Figures 1, 2, 14). As TS is converted to E(Aex_2_), the allosteric unit switches to the α^T^β^T^ state releasing G3P. Synthesis of L-Trp is completed when E(Aex_2_), *via* E(GD_2_), is converted to the E(Ain)(L-Trp) complex followed by the release of L-Trp from the β^T^ conformation of E(Ain) (Figure 14). At this juncture, IGP could bind to the α-site giving the α^R^ conformation, however, the α-site only becomes activated again when the β-subunit is converted to the β^R^ conformation. In this scheme the conversion of β^T^ to β^R^ likely occurs when E(C_1_) is formed via the reaction of E(Ain) with L-Ser to give E(GD_1_) and E(Aex_1_). Since E(C_1_) is only detected as a fleeting intermediate (Drewe and Dunn, 1985), the conformation of E(C_1_) is unknown. It is clear that the quasi-stable E(A-A) has the β^R^ conformation and the α-site is activated. Just as the allosteric switching in hemoglobin is essential to the transport of dioxygen, CO_2_ and H^+^, the allosteric switching in tryptophan synthase is essential for the efficient synthesis of L-tryptophan from IGP and L-serine.

### Active issues concerning allostery and regulation of catalysis in TS

With the wealth of structural and functional data now available, the larger picture of allosteric control in tryptophan synthase is coming into focus. Yet questions remain, particularly on the coupling of structure to the finest level chemical details of the mechanistic transformations. At the chemical level, the linkage between α-site activation and E(A-A) formation is only partially understood. When E(Aex_1_) is converted to E(A-A) *via* E(C_1_), β^T^ is switched to β^R^, the α-site is activated ∼30-fold and the indole sub-site of the β-site is expanded to dimensions tailored to match the VDW dimensions of Indole (Figures 5, 6, 13). This transformation is quite rapid. The β^T^ to β^R^ switch also rearranges the conformation of βAsp305 to form the R state salt bridge with βArg141, and the hydroxymethyl group of the reacting L-Ser moiety of E(Aex_1_) rotates to a position where βLys87 functions as the acid catalyst to facilitate the elimination of the E(C)_1_ hydroxyl to give E(A-A) (Figure 1B) (Holmes et al., 2022). In this transformation, the bonding hybridization of the C_β_ of the reacting substrate switches from sp^3^ to sp^2^, as E(Aex_1_) is converted to E(C_1_). It is not clear if these conformational changes in the site residues and the reacting substrate occur in β^T^ or β^R^. Answering this question will bring into sharper focus an understanding of the linkages between the α- and β-subunit allosteric transitions and the chemical transformations at the α- and β-sites. A second, related issue arises concerning the formation of the indole sub-site in the β-subunit. The indole sub-site of the β-subunit is too small in the β^T^ complexes of E(Ain) and E(Aex_1_) and only expands to dimensions matching those of Indole when β^T^ is switched to β^R^. The mechanism for this allosteric switch remains an elusive and open question, and requires further attention to determine how the allosteric switch to β^R^ triggers the expansion of the indole sub-site to fit the dimensions of indole, and why the sub-site dimensions are linked to the allosteric transition.

## Summary

Allosteric regulation is essential to the efficient utilization of IGP (and therein indole) for the synthesis of L-Trp in enteric bacteria. The integrity of the synthetic pathway is ensured by the protection of both indole and E(A-A) from deleterious side reactions by confining indole to the environs of the α-, and β-subunits and the interconnecting tunnel, sequestering the E(A-A) intermediate from reactive solvent and solute species, and synchronizing the α- and β-reactions to achieve efficiency. This portrait of L-Trp synthesis implies the existence of a strong evolutionary imperative in enteric bacteria to synthesize L-Trp *in situ* rather than depend on the surrounding milieu as a source of L-Trp. In some higher organisms (e.g., maize seedlings), indole is utilized both for the synthesis of L-Trp and as a starting point for the synthesis of a plant secondary metabolite (Kulik et al., 2005). In these divergent synthetic paths, TIM barrel enzyme homologs of the TS α-subunit cleave IGP to indole with quite different regulatory mechanisms. The standalone BX1 homolog in maize has a much greater catalytic activity than the free TS α-subunit, and, in contrast to the TS α-subunit, the structure of BX1 shows a well-ordered loop αL6 with the catalytic Glu residue locked into the postulated active conformation (Kulik et al., 2005).

Consequently, the TS allosteric transitions provide an interesting combination of the switching of a loop between disordered and ordered states in one subunit coupled to a small motion of an 88 amino acid domain in the other subunit of the heterodimeric allosteric unit. This regulation of the final two enzymes in the biosynthesis of L-Trp provides an elegant example of the relation between structure and function in a channeling nanomachine.

## Abbreviations

BZI, Benzimidazole; DOA, dioxindlyl-L-alanine; PLP, pyridoxal-5′-phosphate; E^T^(Ain), internal aldimine (Open, Tense); E^T^(Aex), external aldimine (Open, Tense); E^R^(A-A), α-aminoacrylate (Closed Relaxed); E^R^(C), carbanion (Closed Relaxed); IGP, 3-indole-D-glycerol 3′-phosphate; IPP, 3-indole 3′-propanolphosphate; GP, D,L-α-glycerophosphate; F9, N-(4′-Trifluoromethoxybenzenesulfonyl)-2-aminoethyl Phosphate; F6, N-(4′-Trifluoromethoxybenzoyl)-2-aminoethyl Phosphate; 2AP, 2-aminophenol; 2-HGP, 1-(2′-hydroxyphenylamino) 3-glycerolphosphate; NGP, N-(indolinyl) 3-glycerolphosphate; D-G3P, glyceraldehyde 3-phosphate; OIA, oxindlyl-L-alanine; TS, tryptophan synthase.
